# Systematic in vivo candidate evaluation uncovers therapeutic targets for *LMNA* dilated cardiomyopathy and risk of Lamin A toxicity

**DOI:** 10.1186/s12967-023-04542-4

**Published:** 2023-10-16

**Authors:** Chia Yee Tan, Pui Shi Chan, Hansen Tan, Sung Wei Tan, Chang Jie Mick Lee, Jiong-Wei Wang, Shu Ye, Hendrikje Werner, Ying Jie Loh, Yin Loon Lee, Matthew Ackers-Johnson, Roger S. Y. Foo, Jianming Jiang

**Affiliations:** 1 Department of Biochemistry, Yong Loo Lin School of Medicine, National University of Singapore, Singapore, 117597 Singapore; 2 Centre for Translational Medicine, Cardiovascular Research Institute (CVRI), National University Health System, 14 Medical Drive, Singapore, 117599 Singapore; 3 Cardiovascular Disease Translational Research Programme, NUS Yong Loo Lin School of Medicine, 14 Medical Drive, Level 8, Singapore, 117599 Singapore; 4 Department of Surgery, Yong Loo Lin School of Medicine, National University of Singapore, Singapore, 119228 Singapore; 5 Centre for NanoMedicine, Nanomedicine Translational Research Programme, Yong Loo Lin School of Medicine, National University of Singapore, Singapore, 117609 Singapore; 6 Department of Physiology, National University of Singapore, Singapore, 117593 Singapore; 7 Nuevocor Pte Ltd, 1 Biopolis Drive, Amnios, #05-01, Singapore, 138622 Singapore; 8 A*STAR Skin Research Labs (A*SRL), Agency for Science, Technology and Research (A*STAR), 8A Biomedical Grove, Immunos #06-06, Singapore, 138665 Singapore

**Keywords:** Dilated cardiomyopathy, Lamin A/C, Sun1, Gene therapy, AAV, Fibrosis, Inflammation

## Abstract

**Background:**

Dilated cardiomyopathy (DCM) is a severe, non-ischemic heart disease which ultimately results in heart failure (HF). Decades of research on DCM have revealed diverse aetiologies. Among them, familial DCM is the major form of DCM, with pathogenic variants in *LMNA* being the second most common form of autosomal dominant DCM. *LMNA* DCM is a multifactorial and complex disease with no specific treatment thus far. Many studies have demonstrated that perturbing candidates related to various dysregulated pathways ameliorate *LMNA* DCM. However, it is unknown whether these candidates could serve as potential therapeutic targets especially in long term efficacy.

**Methods:**

We evaluated 14 potential candidates including *Lmna* gene products (Lamin A and Lamin C), key signaling pathways (Tgfβ/Smad, mTor and Fgf/Mapk), calcium handling, proliferation regulators and modifiers of LINC complex function in a cardiac specific *Lmna* DCM model. Positive candidates for improved cardiac function were further assessed by survival analysis. Suppressive roles and mechanisms of these candidates in ameliorating *Lmna* DCM were dissected by comparing marker gene expression, Tgfβ signaling pathway activation, fibrosis, inflammation, proliferation and DNA damage. Furthermore, transcriptome profiling compared the differences between Lamin A and Lamin C treatment.

**Results:**

Cardiac function was restored by several positive candidates (Smad3, Yy1, Bmp7, Ctgf, aYAP1, Sun1, Lamin A, and Lamin C), which significantly correlated with suppression of HF/fibrosis marker expression and cardiac fibrosis in *Lmna* DCM. Lamin C or *Sun1* shRNA administration achieved consistent, prolonged survival which highly correlated with reduced heart inflammation and DNA damage. Importantly, Lamin A treatment improved but could not reproduce long term survival, and Lamin A administration to healthy hearts itself induced DCM. Mechanistically, we identified this lapse as caused by a dose-dependent toxicity of Lamin A, which was independent from its maturation.

**Conclusions:**

In vivo candidate evaluation revealed that supplementation of Lamin C or knockdown of *Sun1* significantly suppressed *Lmna* DCM and achieve prolonged survival. Conversely, Lamin A supplementation did not rescue long term survival and may impart detrimental cardiotoxicity risk. This study highlights a potential of advancing Lamin C and Sun1 as therapeutic targets for the treatment of *LMNA* DCM.

**Graphical Abstract:**

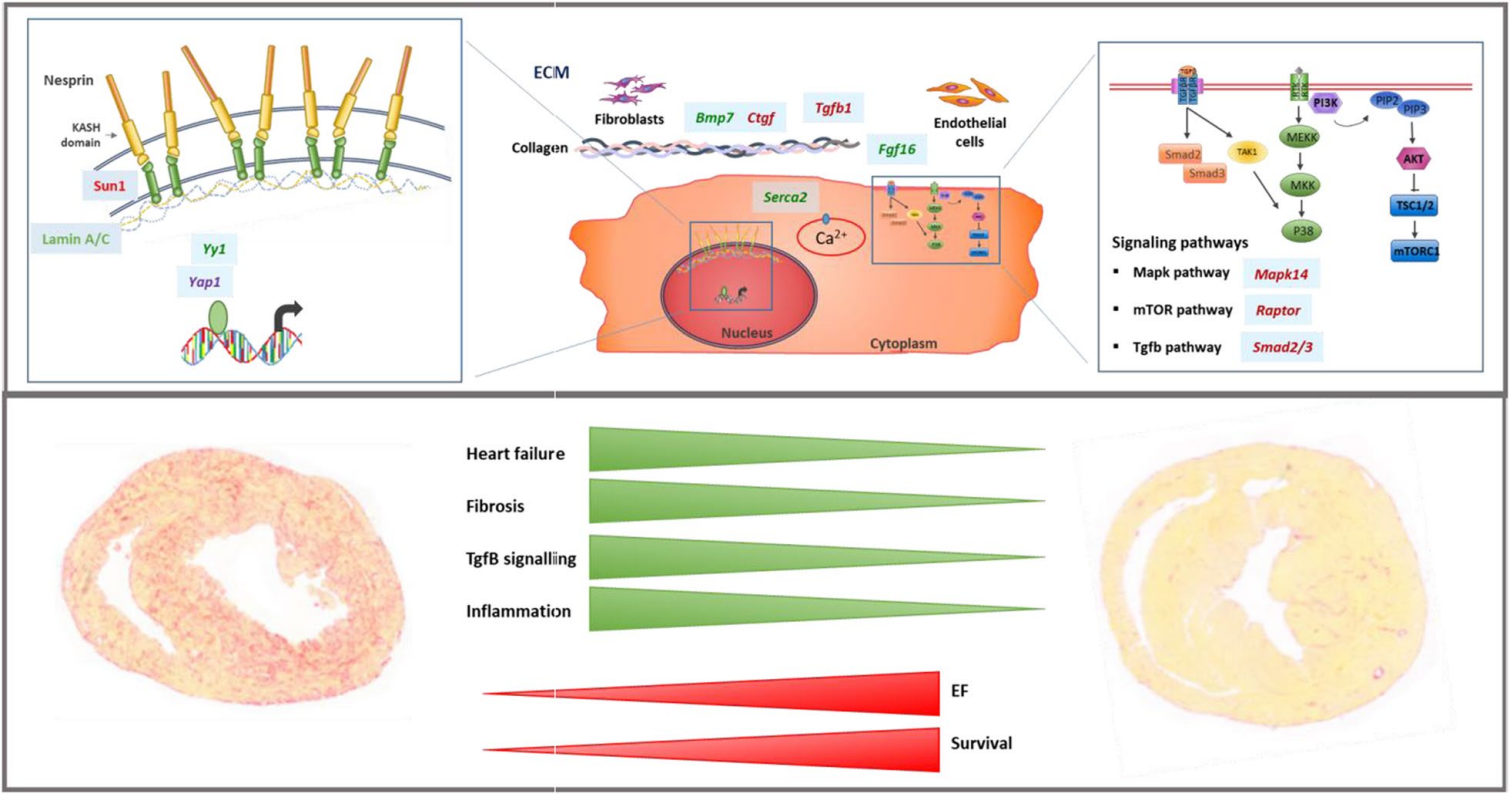

**Supplementary Information:**

The online version contains supplementary material available at 10.1186/s12967-023-04542-4.

## Highlights

- After evaluation of 14 potential candidates in a cardiac-specific *Lmna* DCM model, we demonstrated that *Smad3* shRNA, *Yy1*, combination of *Bmp7* and *Ctgf* (*Bmp7*-*Ctgf* shRNA), *Yap1*, *Sun1* shRNA, Lamin A, and Lamin C improved cardiac function. *Sun1* shRNA and Lamin C particularly prolonged a long-term survival.
- We uncovered that inflammation and DNA damage markers were among the top list of highly correlated markers in addition to traditional HF and fibrosis markers, suggesting these additional markers are important to evaluate the candidates for the treatment efficacy of *LMNA* DCM.
- The study revealed that treating *Lmna* DCM with Lamin A did not work as expected and had toxic effects. The toxicity was found to be dose-dependent and not caused by prelamin A processing.

**Supplementary Information:**

The online version contains supplementary material available at 10.1186/s12967-023-04542-4.

## Background

Dilated cardiomyopathy (DCM) is often caused by genetic pathogenesis, with an estimated prevalence up to 1: 250 [1]. The DCM gene panel containing a list of over 100 genes provides a comprehensive genetic evaluation for patients with a personal or family history of hereditary DCM. Among them, the *LMNA* gene encoding Lamin A and Lamin C is the second most frequently mutated DCM gene [2, 3]. Lamin A is initially expressed as prelamin A and undergoes a series of post-translational modifications to form mature Lamin A [4]. Lamin C arises from alternative splicing. Lamin A and C are identical through their N- termini but diverge at the C-terminus with Lamin C being the shorter isoform, terminating in 6 unique amino acids. Furthermore, as Lamin C does not have the CAAX motif located at the C-terminal end of prelamin A, it lacks a post-translational modification unique to Lamin A.

It is believed that defective function of *LMNA* leads to DCM wherein mechanistic traits and phenotypes are shared in global and especially cardiac specific knockout mouse models [5–7]. Molecular mechanisms underlying *LMNA* DCM have been intensively studied. Currently, there are three main hypotheses for molecular mechanisms underlying *LMNA* related DCM, including dysregulated signaling pathways, lamina-chromatin interactions and lamina-cytoskeleton links [8–10]. Accordingly, many potential candidates have been shown to suppress *LMNA* DCM. Among them, suppression of mTor, Mapk, Pdgf signaling pathways, inhibition of BET bromodomain, or disruption of lamina-cytoskeleton links have been proposed to suppress or ameliorate *LMNA* DCM by using various models including global/cardiac specific knockouts, *LMNA* mutants as well as patient specific iPS derived cardiomyocytes [6, 7, 11–13]. However, it is unknown whether these candidates can effect comparable long term survival, nor have efforts been made to evaluate and compare their efficacy in suppression of *LMNA* DCM. Recently, we produced a cardiac specific knockdown model for *LMNA* DCM (designated as *Lmna* DCM) which is comparable to cardiac specific *Lmna* knockouts [14]. Importantly, this new model does not require intensive crossing to generate compound genetically modified mice.

Autosomal recessive diseases have been successfully treated with recombinant adeno-associated virus (rAAV) mediated gene replacement, exemplified by approval of rAAV9-SMN gene therapy for spinal muscular atrophy (SMA), the leading genetic cause of infant mortality [15]. However, a gene therapy approach for heart failure (HF) was not successful at phase 2 clinical trial due to lack of efficacy, which highlights the importance of prior candidate evaluation [16, 17]. Recently, AAV vector toxicity also emerged in some clinical trials especially at high dosage of virus administration needed for sufficient gene expression and virus distribution [18]. Besides toxicity of AAV vectors, the gene product itself should also be evaluated as, for example, ectopic expression of the *EGFP* reporter induced DCM in a sensitive mouse strain [19]. Apart from recessive diseases, dominant cardiovascular diseases such as cardiomyopathy were also assessed by gene therapy. Proof of principle studies have been attempted by targeting the causal genes of cardiomyopathy including *MHC*, *MYPBC3* and *MYL2* [20–22]. Here, to identify potential targets for the treatment of *LMNA* DCM, we evaluated the efficacy of 14 candidates by modulating their gene expression using rAAV9 in *Lmna* DCM. Lamin A and Lamin C produced by the *Lmna* gene were also included in the assessment since cardiac Lamin A partially rescued global *Lmna* knockouts [23].

## Methods

### Animal protocols

Animal procedures were performed in accordance with the Singapore National Advisory Committee for Laboratory Animal Research guidelines. All mice were housed in animal care facilities and studied using protocols approved by the Institutional Animal Care and Use Committee (IACUC) of National University of Singapore or of the Biological Resource Centre, Agency for Science, Technology and Research. The methods used in the study conformed to the Guidelines on the Care and Use of Animals for Scientific Purposes (NACLAR, Singapore, 2004) as well as the Guide for the Care and Use of Laboratory Animals published by the US National Institutes of Health (NIH Publication, 8th Edition, 2011). Male C57BL/6JINV (Jax) mice were used in this study. *Lmna* cardiac-specific knockout mice were previously described [7]. A power calculation was performed to estimate sample size required to demonstrate significant improvement (error alpha 0.05, power 0.8). Details of virus injection is performed as previously described [14]. For heart harvesting, each mouse was euthanized by cervical dislocation under deep anaesthesia with 5% isoflurane and the heart was exposed by opening of the chest. 15% KCl was then injected into the inferior vena cava to achieve asystole at diastole, and the heart was rapidly isolated. Half of the apex was isolated and immersed it in RNALater (Qiagen, 76104) for RNA extraction. The other half of the apex was snap frozen in liquid nitrogen for protein extraction. For histology experiments, the remaining section of the heart was fixed in 4% paraformaldehyde for 24 h and subsequently embedded in paraffin. A dose of 2.0E13 vg/kg was injection for *Lmna* DCM and 1.0E13 vg/kg was injected for candidates.

### Echocardiogram (Echo)

To measure the cardiac dimensions and function of mice after virus transduction, echocardiography was conducted by using a 40 MHz-550S probe on the VisualSonics Vevo 2100 machine or on a Prospect T1 ultrasound systems from S-Sharp Inc. During the echo and analysis, the researchers were blinded to the animal group allocation and identities. To obtain accurate measurements, LV tracings were averaged from at least three consecutive heartbeats of M-mode, and LVDD (LV diastolic dimensions), LVWT (LV posterior wall thickness), EF (ejection fraction), and FS (fractional shortening) were obtained from short axis images.

### Cell culture and transfection

Transfection of shRNA constructs and other plasmids were done in HEK293T cells (ATCC CRL-1573™, RRID: CVCL_0063). Two transfection methods, PEI (Polysciences. Inc, 24765-2) and Lipofectamine 3000 (Invitrogen L3000015), were used to transfect the cells based on the instructions provided by the manufacturers.

### Recombinant adeno-associated virus (rAAV) production, purification and titration

To produce rAAVs, HEK293T cells were transiently triple transfected with rAAV viral vector with gene, helper plasmid pAdΔF6, and plasmid pAAV2/9 (Penn Vector Core). The viruses were harvested three days post-transfection, and purified using Optiprep density gradient medium (Sigma, D-1556) before being stored at − 80 °C. To determine virus titration, a forward primer (gataaaagcagtctgggctttcaca) and a reverse primer (gagcccatataagcccaagctattg) were used to target the rAAV cTnT promoter region, and quantified by qPCR to determine physical titers.

### Vector construction

For shRNA candidates, primers designed to target 21 base-pair gene-specific regions (Invitrogen) were inserted at the miR-155 backbone of the AAV-*cTnT*-*EGFP* vector. The sequences of shRNAs are as follows:

**Table:** 

*Lmna* shRNA	*agtctcgaatccgcattgaca*
*LacZ* shRNA	*aaatcgctgatttgtgtagtc*
*Raptor* shRNA	*attacagcaagaatgaaggct*
*Tgfb1* shRNA	*ttcctaaagtcaatgtacagc*
*Yap1* shRNA	*tagttccgatccctttcttaa*
*Mapk14* shRNA	*tccactgtctggttatagtgc*
*Smad2* shRNA	*aagagcagcaaattcttggtt*
*Smad3* shRNA	*aatgccagcagggaagttagt*
*Ctgf* shRNA	*cctgtcaagtttgagctttct*
*Sun1* shRNA	*tttggaatgtgttccatggtg*

For vectors used in gain of function studies, *EGFP* in AAV-*cTnT-EGFP* vector was replaced by *Serca*, *Fgf16*, *Bmp7*, *Yy1*, *Lamin A* or *C* (RNAi resistant forms with three silent mutations at the target region)*.* DNSUN1 was obtained by gene synthesis and comprises a human serum albumin signal peptide, the last 453 amino acids of human Sun1, and the KDEL ER-Golgi retrieval signal. DNSUN1 was cloned into a WPRE-containing AAV vector from Addgene (Plasmid #105921, RRID: Addgene_105921) where the CBh promoter was replaced by a cTnT promoter and intron from AAV-*cTnT-EGFP*. AAV-cTnT-3Flag-*hYAP1* S127A (aYAP1) was from Addgene (Plasmid #86558, RRID: Addgene_86558). The cloning primers are as follows:

**Table:** 

*Serca-F*	*ggggctagcatggagaacgctcacacaaagaccg*
*Serca-R*	*gggggtaccttactccagtattgcgggttgttcc*
*Fgf16-F*	*atagctagcatggcggaggtcgggggc*
*Fgf16-R*	*tatgcggccgcttacctatagcggaagagatctctg*
*Bmp7-F*	*ccggctagcatgcacgtgcgctcgctgcgcg*
*Bmp7-R*	*ccgggtaccctagtggcagccacaggcccggac*
*Yy1-F*	*gggcaattgatggcctcgggcgacaccctctacat*
*Yy1-R*	*gggggtacctcactggttgtttttggctttagcg*
*KASH-F*	atagctagcatgcgagccttcctgttccggatcctc
*KASH-R*	tatgcggccgctcagagtggaggaggaccgttggta
*Lamin A-F*	*cccgctagcatggagaccccgtcacagcg*
*Lamin A-R*	*cccggtaccttacatgatgctgcagttctgggagc*
*Lamin C-F*	*cccgctagcatggagaccccgtcacagcg*
*Lamin C-R*	*cccggtacctcagcggcggctgccactca*

### RNAseq library preparation and next generation sequencing

To establish RNAseq libraries, total RNA was extracted from apex of male mice (n = 3 per group). RNA library was prepared using NEBNext® Ultra™ II Directional RNA library prep kit for Illumina (NEB #E7760) according to NEB’s protocol. Libraries were sequenced using the Illumina NovaSeq 6000 sequencing system and paired-end 150 bp reads were generated for analysis. Raw sequencing reads were filtered to remove reads with adapter contamination, those with uncertain and low-quality nucleotides to obtain clean reads. RNAseq data were aligned to GRCm38/mm10 reference genome by using HISAT2 [24]. Following that, a reference-based approached was used to assemble the mapped reads of each sample using StringTie [25]. To count the reads numbers mapped to each gene, FeatureCounts [26] was used. The FPKM (Fragments Per Kilobase of transcript per Million mapped reads) method was used to calculate the expression of each gene. Differentially expressed genes were selected on the basis of adjusted p value (adjP). RNA-seq data was deposit (GSE240745). Potential genetic candidates (adj P < 0.001) were identified from *Lmna* DCM control group compared to Control, *Lamin C* or *Lamin A* treated group. TBtools was used togenerate the volcano plot [27]. The genes that were expressed differently were submitted to Morpheus, where they were subject to Hierarchical clustering and displayed in a heat-map that was color-coded. The Hallmark gene sets were evaluated using Gene Set Enrichment Analysis (GSEA, Broad Institute).

### Quantitative real-time PCR (qPCR)

The levels of transcription were measured using qPCR. Following RNA extraction using Trizol, cDNA was synthesized using *Pure-NA*™ First Strand cDNA Synthesis Kit (Research Instrument, KR01-100). All qPCR was carried out by KAPA SYBR Fast qPCR Master Mix kit (KAPA Biosystems, KR0389) and relative expression levels were quantified using ΔCT. Transcription data were normalized to *Ctcf* expression*.* All qPCR primers are listed as follows:

**Table:** 

Primer	Forward	Reverse
*Nppa*	tttcaagaacctgctagaccacctg	gcttttcaagagggcagatctatcg
*Nppb*	agtcctagccagtctccagagcaat	cgaaggactctttttgggtgttctt
*Myh7*	agcattctcctgctgtttcctt	tgagccttggattctcaaacg
*Col1a1*	gagcctgagtcagcagattgagaac	cctgtctccatgttgcagtagacct
*Col1a2*	acccttctcactcctgaaggctcta	tatgagttcttcgctggggtgttta
*Tgfb1*	gaaggacctgggttggaagtggatc	tgtgttggttgtagagggcaaggac
*Smad 2*	ctctccggctgaactgtctcctact	tccgagtttgatgggtctgtgaagc
*Smad 3*	tccgatgtccccagcacacaataac	ttccggttgacattggacagtaggc
*Atp2a2*	tggtgctgaaaatctccttgcctgt	cataatgagcagcacaaacggccag
*Fgf16*	aactggtacaacacctatgcctcca	catggagggcaacttagaaggatct
*Mapk14*	agctgtgaacgaagactgtgagctc	atgatgcagcccacggaccaaatat
*Sun1*	tatccagaaggagctggaagaaacc	tctctaatagccactcgagggaacc
*Bmp7*	agaatcgctccaagacgccaaagaa	ctctccctcacagtagtaggcagca
*Ctgf*	acacctaaaatcgccaagcctgtca	aatggcaggcacaggtcttgatgaa
*Yy1*	cggggaataagaagtgggagcagaa	caggagggagtttcttgcctgtcat
*Yap1*	gaccctcgttttgccatga	attgttctcaattcctgagac
*Raptor*	tgagtgtcaatggagatgtgcgctt	ccgttgtagatggctgtgaactggt
*KASH*	gccttgtacccatgtcagagaaaga	gttacgtctcgagcatacagccttc
*Ctcf*	atgtcacaccttacctttgcctgaa	ccttcctgctgttcttcctcaaaat
*Lmna*	gaggctcttctcaactccaaggaag	ctgtagcctgttctcagcatccact

### Histological and immunostaining analysis

Heart sample collection and staining were as described previously [14]. Quantification of fibrosis was calculated as the red-stained areas relative to total ventricular area, using NIS-Elements software (Nikon). For antigen retrieval of αSMA, Ki67, Iba1, Yap1, Yy1, cTnI and pH2AX, samples were boiled in citrate buffer (pH 6.0). As for CD3, Sun1 and Nesprin1, samples were boiled in EDTA (pH 9.0) for antigen retrieval. The following primary antibodies were used: αSMA (Sigma, A5228, RRID:AB_262054), Ki67 (Abcam, ab15580, RRID:AB_443209), CD3 (Dako A045201, RRID:AB_2335677), Phospho-Histone H2A.X (Ser139) (Cell Signaling Technology, #9718, RRID:AB_2118009), Iba1 (Wako Laboratory Chemicals (019-19741, RRID:AB_839504), Sun1 (gift from Dr. Brian Burke), YAP1 (Cell Signaling Technology, #14074, RRID:AB_2650491), YY1 (Abcam, ab38422, RRID:AB_778962), Lamin A/C (Cell Signaling Technology, 2032, RRID:AB_10694918), Nesprin1 (Abcam, ab192234, RRID_AB_2917992), PCM1 (Sigma, HPA023374), RRID:AB_1855073 and cTnI (Abcam, ab8295, RRID:AB_306445). DAPI was used for nuclear staining (ThermoFisher, D1306, RRID: AB_2629482). To check for signal specificity, species-specific secondary antibody only controls were used. For each heart sample, investigators unaware of group identities counted the total number of positive signals from three cross-sections, and this data was then standardized to the total number of nuclei or total ventricular area.

### Western blots

Heart tissue collection and western blot were performed as previously described [14]. The following primary antibodies were used: phospho-Smad2 (Ser465/Ser467) (Cell Signaling Technology, 18338, RRID:AB_2798798), phospho-Smad3 (Abcam, ab52903, RRID:AB_882596), Smad 2/3 (Cell Signaling Technology, 5678, RRID:AB_10693547), phospho-p38 (Cell Signaling Technology,9211, RRID:AB_331641), p38 (Cell Signaling Technology, 9212, RRID:AB_330713), phospho-p70S6K (Cell Signaling Technology, 9234, RRID:AB_2269803), p70S6K, (Cell Signaling Technology, 9209, RRID:AB_2269804), phospho-mTOR (Cell Signaling Technology, 5536, RRID:AB_10691552), mTOR (Cell Signaling Technology, 2972, RRID:AB_330978), Lamin A/C (Cell Signaling Technology, 2032, RRID:AB_10694918), Ctgf (Santa Cruz Biotechnology, Inc, sc-365970, RRID:AB_10917259), Yy1 (Thermofsher, PA5-29171, RRID:AB_778962), Bmp7 (Proteintech group, Inc, 12221-1-AP), YAP1 (Cell Signaling Technology, 14074, RRID:AB_2650491), Sun1 (Abcam, ab103021, RRID:AB_2890137), Nesprin1 (Abcam, ab192234, RRID_AB_2917992) and Lamin B1 (Abcam, ab16048, RRID:AB_443298). The secondary antibody used were Donkey anti-Rabbit IgG (H + L) Highly Cross-Adsorbed Secondary Antibody, HRP (Thermofisher, A16035, RRID: AB_2534709) and m-IgGκ BP-HRP (Santa Cruz Biotechnology, sc-516102, RRID: AB_2687626). Protein levels on the blots were detected using the enhanced chemiluminescence system (GE Healthcare, RPN2106) according to the manufacturer’s instructions. Protein band intensity was quantified using Image J (NIH, 1.52e RRID:SCR_003070) and protein levels were normalized to Lamin B1 for mouse hearts.

### Statistical analyses

Prism 10.0.2 (GraphPad Software, La Jolla, California, RRID:SCR_002798) was used to conduct statistical analysis. Normality of sample distribution was assessed by the Shapiro–Wilk normality test. For data with two groups, a two-tailed, unpaired T-test with Welch correction was performed for data that followed a normal Gaussian distribution whereas Mann–Whitney test was performed for data that depart from normality. Brown-Forsythe and Welch ANOVA test with Dunnett’s T3 multiple comparisons were performed for multiple groups. Survival curve was generated according to the Kaplan–Meier method and P value was calculated using Log- rank (Mantel- Cox) test. Quantitative data were shown as mean ± SD. ns, non-significant.

## Results

### *Lmna* gene products suppress *Lmna* DCM

**Fig. 1:**
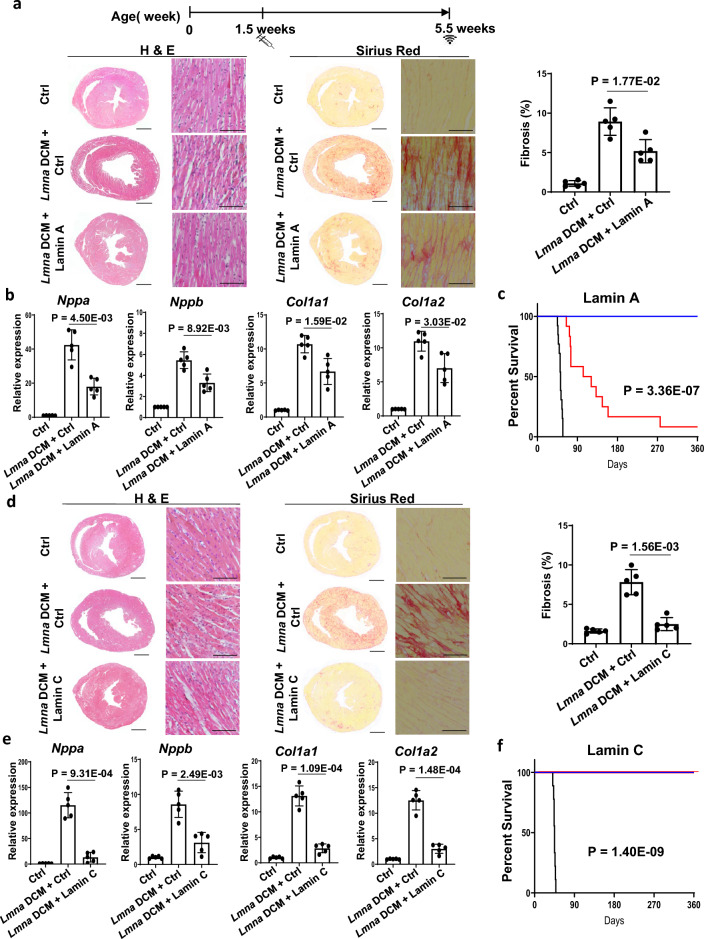
Effect of Lamin A and Lamin C on *Lmna* DCM in mice. (**a** and **d)** Experimental timeline showing timepoints of virus injection and echocardiogram. Sirius Red (SR) and H&E staining of paraffin heart sections and quantifications of *Lmna* DCM mice supplemented with control, *Lamin A* or *Lamin C*. Quantification of myocardial fibrosis of SR sections, n = 5, Brown-Forsythe and Welch ANOVA test with Dunnett’s T3 correction. For complete heart images: magnification = 4 × , scale bar = 1000 µm; for enlarged images: magnification = 20 × , scale bar = 100 µm. (**b** and **e)** Quantitative real-time PCR analyses of *Nppa*, *Nppb*, *Col1a1* and *Col1a2* in *Lmna* DCM mice supplemented with control, *Lamin A* or *Lamin C*, n = 5, Brown-Forsythe and Welch ANOVA test with Dunnett’s T3 correction. (**c** and **f)** Survival curve of *Lmna* DCM mice supplemented with *Lamin A* (Red) or *Lamin C* (Red) compared to *Lmna* DCM mice (Black) and Ctrl (Blue), n = 10, Log- rank (Mantel- Cox) test

To assess whether *Lmna* gene products supress *Lmna* DCM, we administered Lamin A via rAAV9 upon the development of *Lmna* DCM mice. HF markers *Nppa* and *Nppb,* fibrosis markers *Col1a1* and *Col1a2,* fibrosis as well as cardiac function were significantly improved by Lamin A compared to EGFP control (Fig. 1a, b, Table 1). To further assess whether improvement of cardiac function can be translated into long term protection in *Lmna* DCM, we performed survival analysis for Lamin A treatment. Unexpectedly, the protective effect of Lamin A on *Lmna* DCM lapsed starting at 2 months (Fig. 1c). Supplementation with Lamin C, the other product produced by the *Lmna* gene, preserved heart function, supressed HF/fibrosis marker genes, and imparted long-term survival of *Lmna* DCM mice (Fig. 1d–f, Table 1). The difference between Lamin A and Lamin C treatment was not due to excessive overexpression, as Lamin A and Lamin C dose and detection in cardiac tissue were comparable (Additional file 1: Fig. S1).

**Table 1:** Effect of potential candidates on *Lmna* DCM in mice

Age	Virus	N	LVDD	P	LVWT	P	EF%	P	FS%	P
5.5 weeks	*Ctrl*	5	3.93 ± 0.16		0.69 ± 0.06		52.88 ± 4.73		26.86 ± 2.95	
	*Lmna* DCM + *Ctrl*	5	4.30 ± 0.12		0.55 ± 0.05		18.57 ± 1.96		8.32 ± 0.91	
	*Lmna* DCM + Lamin A	5	3.98 ± 0.24	1.34E-02	0.62 ± 0.02	9.66E-02	39.41 ± 4.38	1.91E-04	18.88 ± 2.46	7.61E-04

Age	Virus	N	LVDD	P	LVWT	P	EF%	P	FS%	P
5.5 weeks	*Ctrl*	5	3.93 ± 0.05		0.68 ± 0.03		55.25 ± 3.90		28.34 ± 2.57	
	*Lmna* DCM + *Ctrl*	5	4.27 ± 0.12		0.52 ± 0.03		18.39 ± 2.80		8.24 ± 1.31	
	*Lmna* DCM + Lamin C	5	3.97 ± 0.15	2.01E-02	0.67 ± 0.03	3.20E-04	46.68 ± 3.20	1.20E-06	23.03 ± 2.00	7.03E-06

Echocardiography of *Lmna* DCM mice with Lamin A (top panel) and Lamin C (bottom panel) upregulation at a dose of 1E + 13 vg/kg assessed at 5.5 weeks. P value represents comparisons to *Lmna* DCM, Brown-Forsythe and Welch ANOVA test with Dunnett’s T3 correction. LVDD, left ventricular diastolic dimension; LVWT, LV wall thickness; EF, ejection fraction; FS, fractional shortening

### Difference between Lamin A and Lamin C for the treatment of *Lmna* DCM

**Fig. 2:**
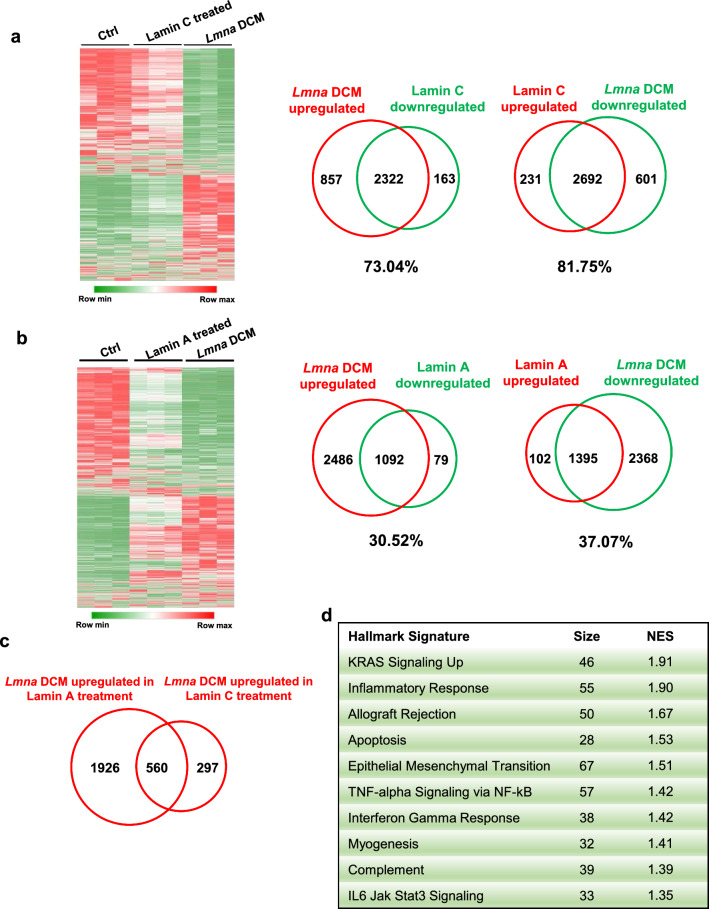
RNAseq analysis of *Lmna* DCM with Lamin A or Lamin C upregulation. (**a** and **b)** Heat map representing color-coded expression level of genes that are significantly changed in *Lmna* DCM mice supplemented with (**a)** Lamin C or (**b)** Lamin A. Venn diagram of overlap between upregulated (red) and downregulated (green) genes in *Lmna* DCM with (**a)** Lamin C or (**b)** Lamin A upregulation compared to *Lmna* DCM mice. Mice were harvested four weeks after transduction, n = 3. (**c)** Venn diagram of overlap between *Lmna* DCM upregulated genes in Lamin A or Lamin C treatment. (**d)** Hallmark signature depicting top 10 dysregulated pathways of upregulated differentially expressed genes (DEGs) which cannot be reversed by Lamin A as designated by GSEA, arranged by NES

To dissect the difference between Lamin A and Lamin C treatment, we compared the transcriptional profiles of various groups including control, *Lmna* DCM, Lamin A treated, and Lamin C treated by RNAseq. Hierarchical clustering compared differentially expressed genes (DEGs, P < 0.001) among the different groups. The Lamin C treated group was closely clustered with the control group, in which up to 80% of DEGs in *Lmna* DCM were reversed by Lamin C (Fig. 2a). In contrast, less than 40% of DEGs in *Lmna* DCM were reversed by Lamin A (Fig. 2b). We further analysed the list of upregulated DEGs that cannot be reversed by Lamin A using GSEA (Fig. 2c, d). Significantly upregulated gene sets identified “Epithelial Mesenchymal transition” as a top enriched set, which is associated with fibrosis. Consistent with this, cardiac fibrosis in *Lmna* DCM was not reduced to a similar level by Lamin A as compared to Lamin C (Fig. 1a, d). Upregulated gene sets were also enriched in “Inflammatory response”, “Allograft rejection” and “TNFa Signaling Via NFkB”, suggesting cardiac inflammation remains activated in Lamin A treated hearts.

### Both Lamin A and its mature form induce DCM in wildtype mice

**Fig. 3:**
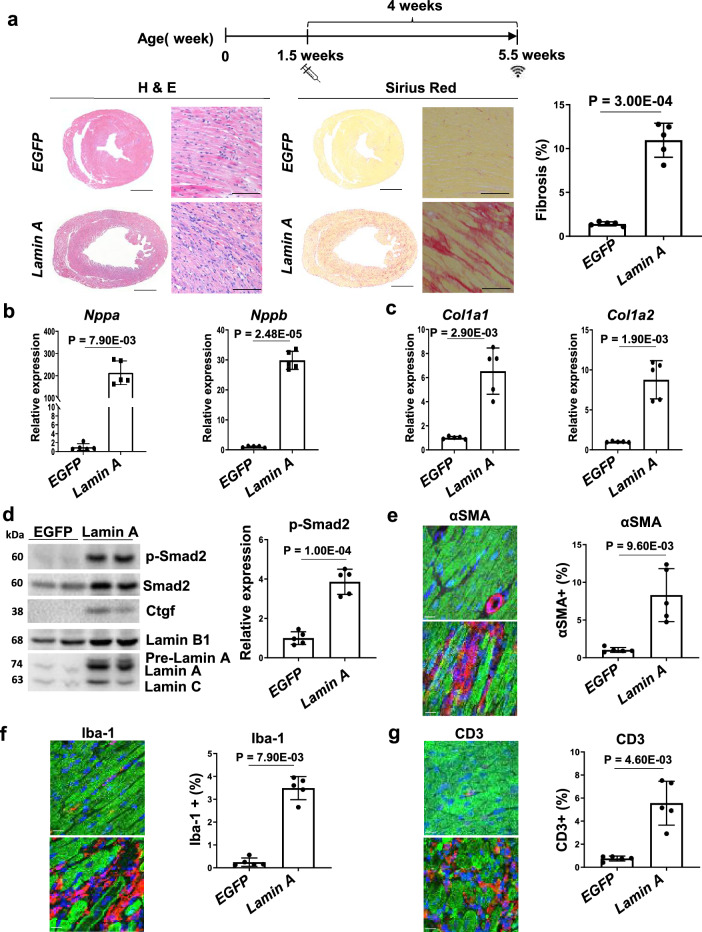
Increased dose of Lamin A in wildtype mice. (**a)** Experimental timeline showing timepoints of virus injection and echocardiogram. SR and H&E staining of paraffin heart Section 4 weeks after transduction of *EGFP* control or Lamin A. Quantification of myocardial fibrosis of SR sections, virus dose, 2.0E + 13 vg/kg, n = 5, two-tailed, unpaired T-test with Welch correction. For complete heart images: magnification = 4 × , scale bar = 1000 µm; for enlarged images: magnification = 20 × , scale bar = 100 µm. (**b, c)** Quantitative real-time PCR analyses of *Nppa*, *Nppb*, *Col1a1* and *Col1a2* in mice transduced with *EGFP* control or *Lamin* *A*, n = 5, two-tailed, unpaired T-test with Welch correction and Mann–Whitney test. (**d)** Western blot and quantitative analysis of phosphorylated Smad2 (p-Smad2) protein levels in mouse heart tissue of mice transduced with *EGFP* control or *Lamin A*, n = 5, two-tailed, unpaired T-test with Welch correction. (**e–g)** Paraffin heart sections (left) and quantifications (right) of (**e)** αSMA (red), (**f)** Iba-1 (red), (**g)** CD3 (red), cTnI (green) and DAPI (blue) positive cells in mice after transduction of *EGFP* control or *Lamin A*, n = 5, two-tailed, unpaired T-test with Welch correction and Mann–Whitney test, scale bar = 50 µm

The impaired protection of Lamin A in *Lmna* DCM and detection of retained fibrosis and inflammatory gene expression by RNAseq analysis, hinted at a detrimental impact of Lamin A following its administration. The specific detrimental effect of Lamin A supplementation was tested by application of an equivalent Lamin A dose to wildtype mice. Although EF was not significantly altered, HF and fibrosis markers, as well as fibrosis were modestly upregulated by Lamin A (Additional file 2: Table S1, Additional file 1: Fig. S2). Since AAV mediated gene delivery is among the most common tools in current gene therapy, it is crucial to dissect the mechanism of this rAAV-Lamin A induced toxicity. An increased dose of Lamin A resulted in significantly impaired cardiac function, enlarged heart chamber of the left ventricle (LV) and reduced LV wall thickness coupled with interstitial fibrosis (Fig. 3a, Additional file 2: Table S2). HF markers *Nppa* and *Nppb* as well as fibrosis markers *Col1a1* and *Col1a2* were significantly upregulated by Lamin A compared to EGFP control (Fig. 3b, c). Consistent with cardiac fibrosis, Lamin A transduced hearts showed a significant upregulation of p-Smad2 and the myofibroblast marker αSMA (Fig. 3d, e). Additionally, Lamin A induced cardiac inflammation is indicated by increased presence of infiltrating Iba-1 + macrophage and CD3 + T cells (Fig. 3f, g). Taken together, these results demonstrate that supplementation of Lamin A by rAAV is detrimental and induces DCM in a dose dependent manner (designated as Lamin A DCM). We noted that accumulation of prelamin A was observed in *Lmna* transduced mice, suggesting that prelamin A is not properly processed upon upregulation of Lamin A by rAAV (Fig. 3d). We hypothesised that this accumulation of prelamin A in CMs could drive detrimental heart function.

Lamin A, initially expressed as prelamin A, undergoes farnesylation and cleavage by Zmpste24 to generate mature Lamin A [28, 29]. The complete processing of farnesylated prelamin A to mature Lamin A is crucial as the accumulation of farnesyl-prelamin A is toxic. Importantly, mice expressing prelamin A only in the absence of Lamin C developed DCM, suggesting that prelamin A accumulation and farneysylation contribute to cardiomyopathy development [30]. To test this possibility, we constructed an AAV vector expressing a mature form of Lamin A, bypassing prelamin A synthesis and farnesylation steps. Transduction of mature Lamin A also led to impaired cardiac contraction, enlarged LV chamber size, reduced LV wall thickness and interstitial fibrosis (Additional file 1: Fig. S3a, Additional file 2: Table S3). Furthermore, we observed a significant increase in HF/fibrosis markers in the mature Lamin A DCM mice compared to controls (Additional file 1: Fig. S3b–e). Consistently, these mice showed significant upregulation of fibrotic mediators as well as cardiac inflammation markers (Additional file 1: Fig. S3f–g). Therefore, upregulation of either pre- or mature Lamin A induced DCM pathogenesis, suggesting that these detrimental effect is caused by the Lamin A product alone and not by impaired prelamin A processing.

Lamin A/C preserves the integrity of the nucleus and DNA. DNA damage is commonly observed in *LMNA* DCM and *Lmna* animal models [31–34]. To further dissect the molecular mechanisms of Lamin A upregulation, we examined DNA damage marker γH2AX in the hearts of Lamin A DCM. Interestingly, γH2AX was significantly increased in the CMs of Lamin A DCM, indicating that Lamin A upregulation induces DNA breaks in mouse hearts and contributes to the detrimental effects of Lamin A on heart function (Additional file 1: Fig. S4).

### Selection of potential candidates for the treatment of *Lmna* DCM

**Fig. 4:**
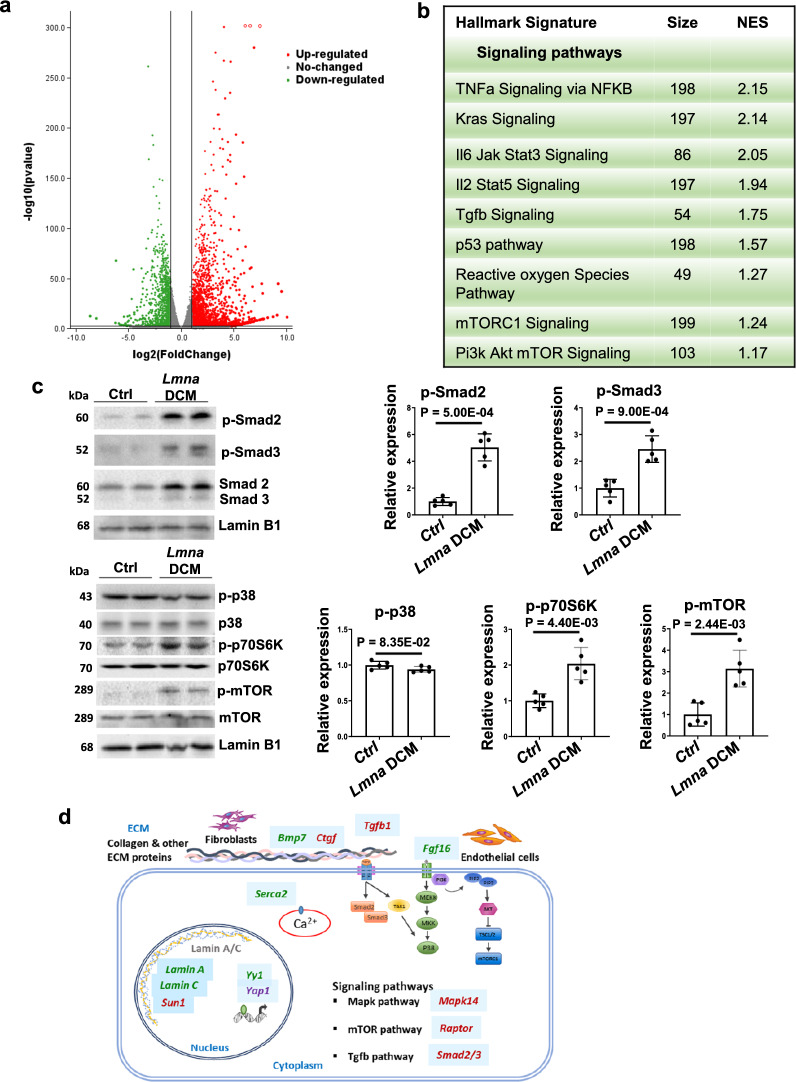
Candidate selection for the treatment of *Lmna* DCM. (**a)** Volcano plot showing differentially expressed genes in heart samples of *Lmna* DCM mice. Downregulated genes are reflected in green and upregulated genes in red. (**b)** Hallmark signature depicting top 10 dysregulated pathways in *Lmna* DCM mice as designated by GSEA, arranged by Normalised Enrichment Score (NES). (**c)** Western blot and quantitative analysis of phospho-Smad2, phospho-Smad3, phospho-p38, phospho-p70S6K and phospho-mTOR protein levels in mouse heart tissues of control or *Lmna* DCM mice, n = 5, two-tailed, unpaired T-test with Welch correction. (**d)** Schematic diagram of selected candidates in the cytoplasm and nucleus of cardiomyocyte as well as extracellular matrix (ECM)

*LMNA* gene replacement is able to treat *LMNA* DCM caused by haploinsufficiency through compensation for the loss of functional *LMNA*. However, *LMNA* DCM caused by dominant negative mutations are not amenable to gene replacement. To evaluate other potential candidates, we analysed our generated RNAseq data for *Lmna* DCM. Significant dysregulated genes were detected by Gene Set Enrichment Analysis (GSEA, Broad Institute) (Fig. 4a, b). Dysregulated gene sets identified hallmark signature associated signaling pathways including KRas, Tgfb and mTorc1, many of which have been shown to be deregulated in various *LMNA* related models. To validate these pathways in *Lmna* DCM, we examined the key factors in these pathways in heart tissues by Western blot. Consistent with previous studies, we observed that Tgfβ and mTor signaling pathways were dysregulated in *Lmna* DCM [11, 35] (Fig. 4c). However, the p-p38/Mapk pathway did not change significantly. Based on our pathway analysis/validation as well as previous studies, we selected to evaluate candidates including *Tgfb1*, *Smad2*, *Smad3* for Tgfβ signaling pathway, *Rapto*r for mTor signaling pathway, *Fgf16* and *Mapk14* for Mapk signaling pathway, *Serca2* for calcium handling and *Sun1* for the linker of nucleoskeleton and cytoskeleton (LINC). Our recently studied genes such as *Bmp7*, *Ctgf*, *Yy1*, *Yap1* were also included in the comparison [14, 36] (Fig. 4d). We further examined their gene expression in heart tissue of *Lmna* DCM. The expression of *Tgfb1*, *Fgf16*, *Bmp7*, *Ctgf*, *Serca2*, *Smad2*, *Raptor* and *Yap1* were dysregulated in *Lmna* DCM. Other signalling downstream molecules or transcriptional regulators such as *Smad3*, *Mapk14*, *Yy1* and *Sun1*, were not transcriptionally regulated in *Lmna* DCM (Additional file 1: Fig. S5).

### Positive candidates extend survival of *Lmna* DCM

**Fig. 5:**
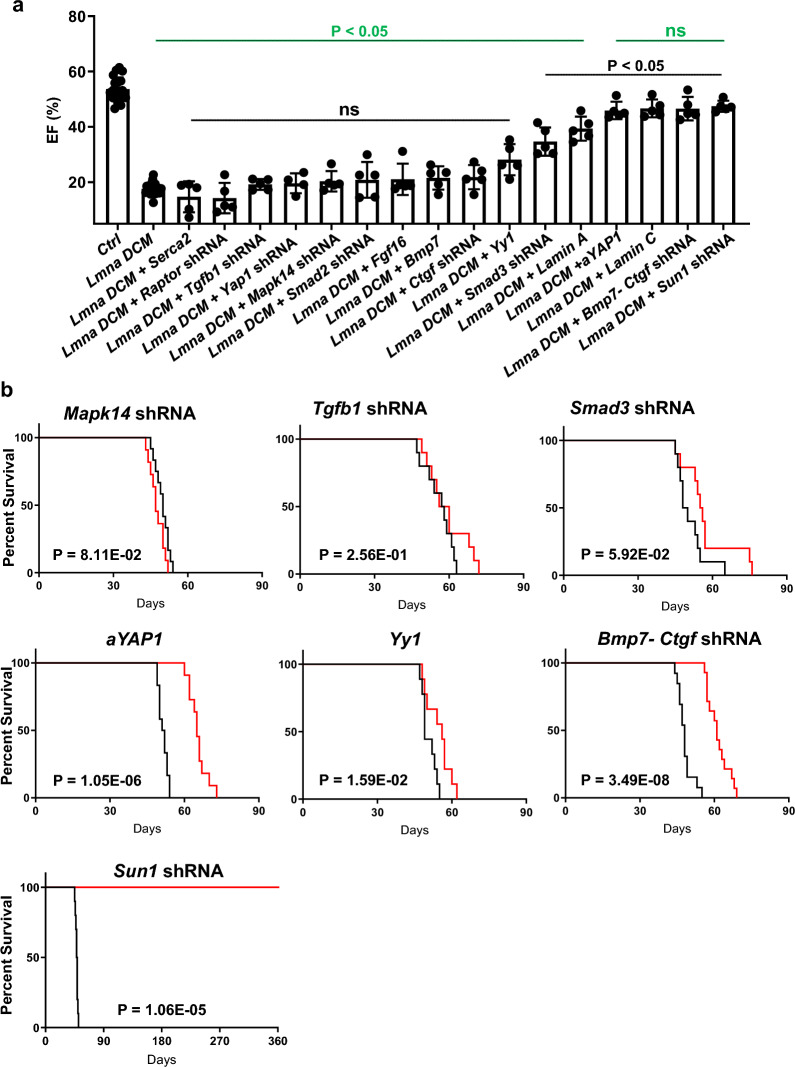
Positive candidates extend the survival of *Lmna* DCM. (**a)** Comparison of EF (%) assessed by echocardiography in control and *Lmna* DCM mice supplemented with potential candidates compared to control (P value in green) or *Lmna* DCM (P value in black). Echocardiography was performed 3 weeks after transduction, n = 5, Brown-Forsythe and Welch ANOVA test with Dunnett’s T3 correction. (**b)** Survival curve of *Lmna* DCM mice supplemented with selected candidates (Red) compared to *Lmna* DCM mice (Black), n = 10, Log- rank (Mantel- Cox) test

To evaluate the functions of these selected candidates in *Lmna* DCM, we modulated their expression by rAAV9. The knockdown efficacy in vitro was assessed by a 2-color system developed previously [37] (Additional file 1: Fig. S6). At least 75 percent knockdown was achieved for loss-of-function candidates. All candidates were assessed in *Lmna* DCM. The effects of each candidate on cardiac function were compared using echocardiography (Fig. 5a, Additional file 2: Table S4). These candidates alone did not affect cardiac function and survival of wildtype mice in the observed time window (Additional file 2: Table S5, Additional file 1: Fig. S7). Among them, *Smad3* shRNA, *Yy1*, combination of *Bmp7* and *Ctgf* shRNA (*Bmp7-Ctgf* shRNA), *a*YAP1, *Sun1* shRNA, Lamin A, and Lamin C, can significantly improve the ejection fraction (EF) in *Lmna* DCM. Their upregulation or knockdown efficacy in vivo was validated by Western Blot (Additional file 1: Fig. S8). Selected candidates including *Smad2*, *Tgfb1, Mapk14*, *Smad3*, *Yy1*, *Yap1* and *Sun1* were included to assess the effect of dosage on gene expression. Echocardiography results showed that increased dosage of the selected candidates did not change their effect on cardiac function (Additional file 2: Table S6). In addition, the knockdown and overexpression efficacy of *Sun1*, *Yap1* and *Yy1* at different dosages was examined by immunostaining (Additional file 1: Fig. S9 and S10). To further assess whether improvement of cardiac function can be translated into long term protection in *Lmna* DCM, we performed survival analysis for all positive candidates (Fig. 5b). Two negative candidate shRNAs *Tgfb1* and *Mapk14* were included for comparison. Consistently, knockdown of these two negative candidates did not prolong survival for *Lmna* DCM. Although cardiac EF in *Lmna* DCM was significantly improved to ~ 35% upon *Smad3* shRNA treatment, the median survival was not significantly extended, suggesting improved cardiac function does not always extend to prolonged animal survival. *Yy1*, *a*YAP1 or *Bmp7-Ctgf* shRNA extended the median survival of *Lmna* DCM by a modest 15–30%. Strikingly, *Sun1* shRNA extended survival of *Lmna* DCM by at least tenfold. Importantly, cardiac function in *Lmna* DCM was preserved by *Sun1* shRNA, similar to Lamin C (Additional file 1: Fig. S11). As a key component of the Linker of Nucleoskeleton and Cytoskeleton (LINC) complex, Sun1 plays a pivotal role in linking the nuclear lamina to the cytoskeleton by bridging nuclear lamins with cytoskeleton-interacting KASH domain proteins [9].

To dissect the roles of *Sun1* in *Lmna* DCM, we first assessed *Sun1* expression and protein levels. No significant change of *Sun1* was detected in *Lmna* DCM compared to that in control mice (Additional file 1: Fig. S5, S9a and S12a). Similar to Lamin A/C, Sun1 signal was detected around the nuclei in control cardiac cells (Additional file 1: Fig. S12b). Interestingly, we observed the number of cardiomyocyte (CM) nuclei with abnormal Sun1 distribution was significantly increased in *Lmna* DCM compared to those in the controls (Additional file 1: Fig. S12b), suggesting that *Lmna* knockdown perturbs Sun1 distribution. Analysis of the nuclear shape of CM with abnormally distributed Sun1 revealed a significant increase of nuclear protrusion when compared to CM with normal Sun1 distribution (Additional file 1: Fig. S12c). Importantly, the number of nuclei with abnormal nuclear protrusion in *Lmna* DCM mice was significantly reduced by *Sun1* shRNA (Additional file 1: Fig. S12c), indicating that knockdown of *Sun1* preserved nuclear integrity of the CMs in *Lmna* DCM.

Nesprins interact with Sun1 via their conserved KASH domain [38]. To further elucidate the role of LINC complex in *Lmna* DCM, we investigated Nesprins and found no significant changes in *Syne1* (coding for Nesprin-1) expression and protein levels (Additional file 1: Fig. S13a, b). Sun1 and Nesprin-1 were co-localized in the nucleus (Additional file 1: Fig. S13c). Interestingly, we observed a significant increase of abnormal Nesprin-1 distribution in CM nuclei in *Lmna* DCM compared to controls (Additional file 1: Fig. S13d), indicating that Lamin A/C knockdown also induces abnormal distribution of Nesprin-1. Furthermore, the proportion of nuclear protrusion with abnormally distributed Nesprin-1 was significantly increased compared to those with normal Nesprin-1 distribution in *Lmna* DCM CMs (Additional file 1: Fig. S13d).

Our earlier work suggested that mutating the KASH domain of Nesprin-1 could ameliorate *Lmna* DCM [39]. To perturb the interaction between Sun1 and Nesprins in *Lmna* DCM mice, we introduced the KASH domain of Nesprin-1 to compete for binding with Sun1. Interestingly, upregulation of the KASH domain suppressed *Lmna* DCM. The EF of *Lmna* DCM mice was significantly improved by KASH domain expression (Additional file 2: Table S7). Furthermore, cardiac fibrosis was significantly reduced in *Lmna* DCM mice with KASH domain upregulation (Additional file 1: Fig. S14a, b). Consistently, we observed a significant decrease in HF markers *Nppa* and *Nppb* as well as fibrosis markers *Col1a1* and *Col1a2* with KASH treatment (Additional file 1: Fig. S14c). Taken together, these results indicate that the LINC complex is a key target for treatment of *Lmna* DCM.

Consecutive AAV delivery into the same mouse is not possible due to the development of neutralizing antibodies to AAV capsids following initial AAV exposure. To assess whether potential candidates can halt or reverse the DCM phenotype, alternative *Lmna* DCM models other than AAV9-*Lmna* shRNA are required. Previously, we established an inducible *Lmna* DCM mouse model by CM-specific *Lmna* deletion in CMs in vivo. Disruption of the LINC complex by AAV9-mediated expression of a dominant negative SUN1 (DNSUN1) construct suppressed *Lmna* DCM and resulted in an increased lifespan in this DCM model [7]. A follow-up study showed that AAV9-DNSUN1 can suppress inducible *Lmna* DCM 2.5 weeks after induction of DCM, when EF has begun to decline (Additional file 2: Table S8). Importantly, the lifespan of inducible *Lmna* DCM mice was significantly prolonged, demonstrating that LINC complex disruption is a potential treatment, and not just a prophylaxis, for *Lmna* DCM (Additional file 1: Fig. S15a). However, whether expression of DNSUN1 or the KASH domain, or silencing Sun1 to disrupt the LINC complex, or overexpression of Lamin C, can suppress fully developed and late stage *Lmna* DCM requires further optimization due to the quick development of the phenotype and rapid mortality of our currently used mouse models.

### Additional markers are required to evaluate candidates for the treatment of *Lmna* DCM

**Fig. 6:**
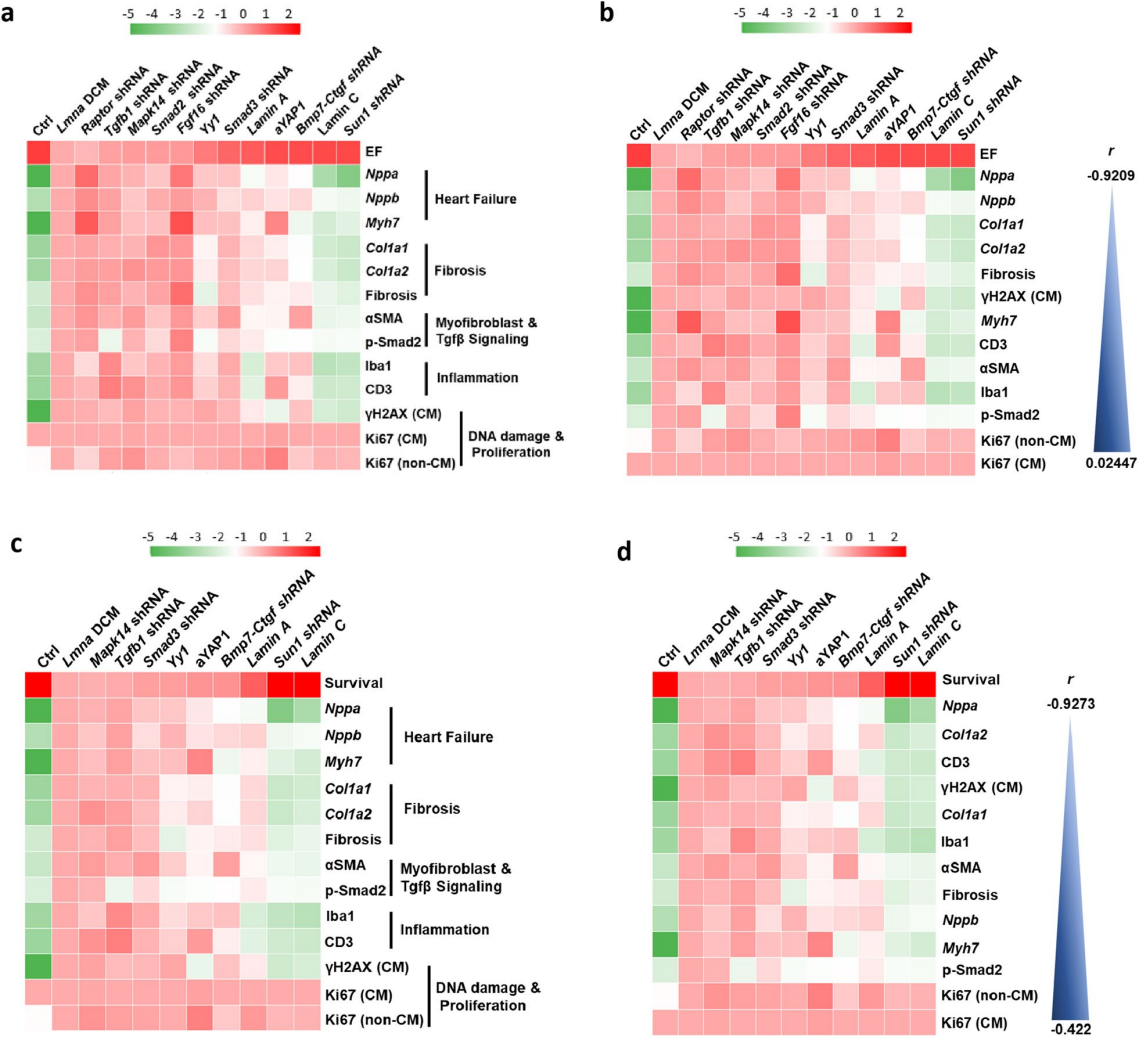
Evaluation of disease markers in *Lmna* DCM. (**a** and **c)** Heat map representing color-coded log_2_ fold change of the gene expression of *Nppa*, *Nppb*, *Myh7*, *Col1a1* and *Col1a2*, immunostaining (αSMA, Iba1, CD3, γH2AX, Ki67), Western blot (p-Smad2) and histology (fibrosis). (**a)** EF or (**c)** survival of *Lmna* DCM mice supplemented with selected candidates or control, n = 5. (**b** and **d)** Hierarchical clustering and correlation analysis of log_2_ fold change of selected markers with (**b)** EF or (**d)** survival of *Lmna* DCM mice supplemented with selected candidates or control by nonparametric Spearman correlation, n = 5

For a comprehensive understanding of whether the candidates and markers were related to the treatment of *Lmna* DCM, we aligned EF with commonly used markers including *Nppa* and *Nppb* for HF markers, and *Cola1* and *Cola2* for fibrosis related markers (Fig. 6a). Additionally, we incorporated picrosirius red staining for fibrosis level, αSMA staining for myofibroblast activation, p-Smad2 level for the activation of Tgfβ signaling pathway, Iba1 and CD3 staining for the infiltration of macrophages and T cells, γH2AX staining for DNA damage, and Ki67 staining for cardiac cell proliferation. Most markers except Ki67 were negatively correlated with cardiac function (Spearman’s correlation, − 0.640 ~ − 0.921, P < 0.05–0.001). HF and fibrosis markers as well as fibrosis level ranked closely with EF (Fig. 6b). However, these commonly used markers in most HF studies were not sufficient to predict the long-term efficacy for the treatment. The lapses of protective effect of the candidates were associated with an increase of markers including γH2AX, Iba1 and CD3. We further analyzed the correlation of the markers with animal survival for the treatment of *Lmna* DCM (Fig. 6c). Similar to EF, most markers except Ki67 and p-Smad2 were negatively correlated to animal survival (Spearman’s correlation, − 0.782 ~ − 0.927, P < 0.05–0.001). In addition to HF and fibrosis markers, top ranking markers included immune (CD3) and DNA damage markers (γH2AX) which had a highly negative correlation with animal survival, suggesting that these additional markers are important to evaluate candidates for the treatment of *Lmna* DCM (Fig. 6d).

## Discussion

We have systematically evaluated candidates related to key pathways in *Lmna* DCM including Tgfβ/Smad, Fgf/Mapk and mTor. Among them, the Tgfβ/Smad signaling pathway is commonly activated in *Lmna* DCM as well as many other HF models [40]. Smad3 plays an essential role in heart remodelling in MI or TAC-induced heart failure models, although it plays different roles in CFs and CMs [41–43]. Consistent with MI or TAC models, CM specific reduction of *Smad3* ameliorated the impaired cardiac function in *Lmna* DCM mice. However, cardiac fibrosis, markers for HF/fibrosis and activation of myofibroblasts were only modestly reduced. Critically, this protective effect quickly lapsed, resulting in no significant improvement of survival of *Lmna* DCM mice. p38α MAP kinase and mTor signaling pathways are activated in *Lmna*^H222P/H222P^ or *Lmna* -/- mouse models. Inhibition of these pathways by p38α inhibitor ARRY-371797 or mTOR inhibitor rapamycin prevents cardiac dilation, improves cardiac function and/or modestly extends survival of diseased mice [11, 44, 45]. However, knockdown of *Mapk14* (coding for p38α), *Raptor* (a key component for mTORC1 signaling) or overexpression of *Fgf16* (a downstream gene of *Gata4*) specifically in CMs did not significantly suppress *Lmna* DCM, suggesting their protective roles might be contributed by non-CMs or are specific to certain disease models [46]. Moreover, modulation of both *Bmp7* and *Ctgf*, the secreted factors regulated by *Yy1*, improved cardiac function and extended survival by ~ 30%. However, *Lmna* DCM mice died quickly in a short time window after treatment, suggesting modulation of signaling pathways has a broad but modest therapeutic benefit for *Lmna* DCM. Similarly, modulation of calcium handling and proliferation related genes has a limited beneficial role in *Lmna* DCM.

We evaluated Lamin A, Lamin C and the LINC complex component Sun1 as therapeutic candidates for *Lmna* DCM. These three proteins are co-localized and interact in the nuclear envelope. Lamin A and Lamin C are the products of the *Lmna* gene via alternative splicing. Lamin A/C forms a matrix to preserve the strength and integrity of CM nuclei. Although Lamin A has a unique C-terminal modification and maturation process compared to Lamin C, Lamin A and Lamin C appear to be functionally equal and interchangeable because Lamin A or Lamin C only knockout mice are normal in general [47, 48]. Prelamin A only transgenic mice instead succumbed to DCM [30]. Consistent with Lamin C only transgenic mice, supplementation of Lamin C suppressed *Lmna* DCM and achieved long term survival of at least one year. Although Lamin A supplementation also prolonged survival, this protection lapsed, with median survival limited to 110 days. We identified dose-dependent Lamin A toxicity as the novel pathological mediator of this effect. This is inconsistent with previous results that transgenic mice expressing Lamin A alone in the absence of Lamin C had no disease phenotypes [48]. One possible explanation for this discrepancy is that rAAV9 mediated upregulation of transgenes is acute and rapid in comparison to classic transgenic techniques. In addition, we speculate that there is a need to maintain a specific ratio between Lamin A and Lamin C levels in the CMs, given that endogenous Lamin C is the dominant form in CM compared to Lamin A [14]. It is possible that Lamin A upregulation, resulting in an increased Lamin A/C ratio, led to detrimental effects. Hence, the necessity to ensure a careful balance of cellular Lamin A levels has important implications for *LMNA* DCM gene therapy approaches. We note that this is consistent with a reported higher frequency of misshapen nuclei in mouse embryonic fibroblasts of Lamin A only mice [48]. Lamin A upregulation induced DNA damage, however, the exact mechanisms of how Lamin A upregulation resulted in DCM is yet unknown and warrants further study. Sun1 interacts with Lamin A/C, linking nucleoskeleton to cytoskeleton via KASH domain proteins such as Nesprin-1. Perturbing this interaction via *Sun1* shRNA or KASH overexpression significantly suppressed *Lmna* DCM and achieved long term survival, which is consistent with a protective role of dominant negative Sun1 in a DCM model induced by cardiac specific *Lmna* knockout [7]. *Sun1*-/- or mutating the KASH domain of Nesprin-1 also ameliorates *Lmna* null and progeroid *Lmna*^Δ9^ mutant mice, suggesting that Sun1/Nesprin-1-containing LINC complexes can serve as a therapeutic target for other *Lmna* related diseases [39, 49, 50]. A comparable beneficial effect between *Sun1* knockdown and Lamin C gene replacement for *Lmna* DCM advances a potential of translational research for *LMNA* DCM treatment. In addition, this finding implies that perturbing the LINC complex can achieve a similar efficacy as gene replacement for *Lmna* DCM, which can be potentially extended to the treatment for *LMNA* DCM caused by different pathological variants. To date, more than 400 *LMNA* mutations have been described (http://www.umd.be/LMNA/) and among them, 165 unique mutations distributed along the entire *LMNA* gene have been linked to cardiomyopathy [51]. Missense mutations in *LMNA* have been proposed to act mainly through a dominant negative pathway while truncating variants likely result in haploinsufficiency [52, 53]. Only a handful of mutations are unique to Lamin A, the majority of which are located on the C terminus of prelamin A, a hotspot for progeria where DCM mutations are rare [51, 52, 54] (Additional file 1: Fig. S16). Hence, Lamin C gene replacement is suitable for the treatment of *LMNA* DCM with haploinsufficiency mechanism through compensation for the loss of functional Lamin A/C in which the majority of mutations affect both Lamin A and C. On the other hand, the rescue role of *Sun1* knockdown, KASH overexpression or DNSUN1 overexpression will extend to *LMNA* DCM caused by dominant negative mutations.

We compared various pathological markers to heart function and animal survival. Heart function was negatively correlated with the expression of HF/fibrosis markers as well as fibrosis which are utilised commonly in most HF related research. Importantly, tests for B-type natriuretic peptide (BNP, coded by *NPPB*) and N-terminal pro-B-type natriuretic peptide (NT-proBNP) are extensively utilized as diagnostic and prognosis biomarkers for HF in clinical settings due to their highly sensitivity and specificity. However, these common markers alone cannot predict the long-term efficacy of treatments for *Lmna* DCM. Activation of resident or infiltration of blood-derived immune cells are involved in pathological inflammatory pathways and tissue reparative processes in HF [55–57]. Upregulation of inflammatory cytokines contributes to the pathogenesis of *LMNA*-cardiomyopathy patients and affects the severity of cardiac phenotype [58]. Reduced immune cell numbers correlated with long term animal survival following treatment by *Sun1* shRNA or Lamin C supplementation in *Lmna* DCM. Whether other types of immune cells, resident or infiltrated are also related to heart function and animal survival requires further investigation. DNA damage was observed in CMs of *Lmna* DCM as well as *LMNA* mutant models. Suppression of DNA damage was also highly correlated with animal survival, suggesting that maintenance of DNA/nuclei integrity is important for long term efficacy of *Lmna* DCM therapy.

In this study, we used rAAV9 as well as a CM specific promoter to modulate gene expression specifically in CMs. Besides cardiac tropism, AAV9 has a broad tropism to other organs including neuron, liver and lung [59]. AAV9’s liver tropism also facilitates CRISPR-based gene therapy for liver targets related to cardiovascular disease including atherosclerotic cardiovascular disease (PCSK9) [60]. However, AAV9 has limited access to cardiac fibroblasts, smooth muscle cells, and endothelial cells which impedes application to non-CMs in hearts. Recently AAV9 capsid modification enhanced potency for skeletal muscle which could extend applications to muscular disease such as Duchenne muscular dystrophy [61]. Since our *Lmna* DCM model is also cardiac specific, the evaluation of selected candidates specifically in CMs highlights a potential of advancing positive candidates to the treatment of cardiac specific *LMNA* DCM [62]. Whether these candidates also benefit other *LMNA* related disease requires further validation.

We found that Lamin C and the LINC complex are promising gene therapy targets for *LMNA* DCM. However, this study has several limitations. First, we only modulate *Lmna* and potential candidates in CMs. Hence, we may have overlooked the functions and contribution of these candidates in non-CMs. Second, due to the limitations of rAAV9 delivery, we were unable to obtain satisfactory expression levels via consecutive virus injection to assess whether Lamin C and *Sun1* shRNA can reverse established *Lmna* DCM. To overcome these limitations, alternative animal models such as inducible knockout or knockin mice can be used to further evaluate the potential candidates. Finally, atrioventricular block and arrhythmias frequently occur in patients with *LMNA*-associated DCM. It is worth evaluating whether modulation of Lamin C and Sun1 are able to suppress these abnormal ECG features.

Here, we evaluated 14 potential therapeutic candidates in *Lmna* DCM. Heart function, animal survival and pathological cardiac markers were analyzed and compared, revealing Lamin C and Sun1 as viable therapeutic targets, and an unforeseen risk of Lamin A toxicity. This study further provides a simple platform that can be adapted to evaluate and compare other potential candidates for *LMNA* DCM.

## Conclusions

This study demonstrates that gene replacement or LINC complex perturbation has a profound and beneficial impact on *LMNA* DCM, providing a solid foundation for the therapy development. Currently, researchers are investigating AAV-mediated gene therapy approaches for cardiovascular disease and their potential benefits for HF patients. These findings suggest that gene therapy could be a potential option for the treatment of *LMNA* DCM.

### Supplementary Information

**Additional file 1: Figure S1.** Upregulation of Lamin A and Lamin C in *Lmna* DCM mice. Evaluation of the upregulated level of Lamin A and Lamin C *in vivo* by western blot in mouse whole heart tissue lysates of control or *Lmna* DCM groups with control, Lamin A or Lamin C upregulation. **Figure S2.** Upregulation of Lamin A in mice. (**a)** Experimental timeline showing timepoints of virus injection and echocardiogram. Cardiac performance was assessed by echocardiogram at 5.5 weeks-old. SR and H&E staining of paraffin heart sections 3-4 weeks after transduction of *EGFP* control or Lamin A. Quantification of myocardial fibrosis of SR sections, virus dose, 1.0E+13 vg/kg, n = 5, Mann-Whitney test. For complete heart images: magnification = 4 ×, scale bar =1000 µm; for enlarged images: magnification = 20 ×, scale bar = 100 µm. (**b, c)** Quantitative real-time PCR analyses of *Nppa*, *Nppb*, *Col1a1* and *Col1a2* in mice transduced with *EGFP* control or Lamin A, n = 5, two-tailed, unpaired T-test with Welch correction and Mann-Whitney test. **Figure S3.** Upregulation of mature Lamin A by AAV leads to DCM and cardiac fibrosis. (**a)** SR and H&E staining of paraffin heart sections and quantifications of mice transduced with *EGFP* control or mature Lamin A. Quantification of myocardial fibrosis of SR sections, virus dose, 2.0E+13 vg/kg, n = 5, two-tailed, unpaired T-test with Welch correction. For complete heart images: magnification = 4 ×, scale bar =1000 µm; for enlarged images: magnification = 20 ×, scale bar = 100 µm. (**b, c)** Quantitative real-time PCR analyses of *Nppa*, *Nppb*, *Col1a1* and *Col1a2* in mice transduced with *EGFP* control or mature Lamin A, n = 5, two-tailed, unpaired T-test with Welch correction. (**d)** Western blot of p-Smad2 protein levels in mouse heart tissues of mice transduced with *EGFP* control or mature Lamin A, n = 5, two-tailed, unpaired T-test with Welch correction. (**e–g)** Paraffin heart sections (left) and quantifications (right) of (**e)** αSMA (red), (**f)** Iba-1 (red), (**g)** CD3 (red), cTnI (green) and DAPI (blue) positive cells in mice after transduction of *EGFP* control or mature Lamin A, n = 5, two-tailed, unpaired T-test with Welch correction, scale bar = 50 µm. **Figure S4.** DNA damage levels in Lamin A DCM. (**a)** Representative images (left) and quantifications (right) of paraffin heart sections with immunostaining with for γH2AX (red), cTnI (green) and DAPI (blue), n = 5, two-tailed, unpaired T-test with Welch correction, scale bar = 5 µm. Arrow indicates γH2AX-positive CMs in Lamin A DCM. **Figure S5.** Expression levels of selected candidates in *Lmna* DCM. Quantitative real-time PCR analyses of *Tgfb1*, *Smad2*, *Smad3*, *Atp2a2*, *Fgf16*, *Mapk14*, *Sun1*, *Bmp7*, *Ctgf*, *Yy1*, *Yap1* and *Rptor* expressions in control or *Lmna* DCM mice, n = 5, two-tailed, unpaired T-test with Welch correction and Mann-Whitney test. **Figure S6.** Knockdown efficacy of RNAi in vitro. 2-color system and quantification of shRNA knockdown efficacy of selected candidates. HEK293T cells co-transfected with selected candidates (green) and corresponding shRNAs or control shRNA (Red), n=4, two-tailed, unpaired T-test with Welch correction. Magnification = 4 ×, scale bar=500 µm. **Figure S7.** Evaluation of Survival Rate for selected candidates in wildtype mice. Survival curve of wildtype mice supplemented with Ctrl shRNA (black), *EGFP* Ctrl (black) or selected candidates (red), n = 10. **Figure S8.** Upregulation level and RNAi efficacy *in vivo* for positive candidates. Evaluation of the upregulated level of positive candidates Yy1, Bmp7 and aYAP1 and RNAi knockdown efficacy of positive candidates Smad3, Ctgf and Sun1 *in vivo* by western blot in mouse heart tissues of control or *Lmna* DCM groups. * indicates a non-specific band. **Figure S9.** Different dose of AAV9-shRNA. (**a** and **b)** Paraffin heart sections of (left) of (**a)** Sun1 (red), (**b)** Yap1 (red), cTnI (green) and DAPI (blue) and quantifications (right) of (**a)** Sun1 intensity (red), (**b)** Yap1 intensity (red) after transduction of 1E+13 vg/kg or 2E+13 vg/kg dose for (**a)** *Sun1* shRNA or (**b)** *Yap1* shRNA in control or *Lmna* DCM mice, n = 5, Brown-Forsythe and Welch ANOVA test with Dunnett’s T3 correction, scale bar = 10 µm. Arrows indicate Sun1 (**a)** or Yap1 (**b)** positive CMs. **Figure S10.** Different dose of AAV9 overexpression. (**a **and **b)** Paraffin heart sections of (left) (**a)** Yap1 (red), (**b)** Yy1 (red), cTnI (green) and DAPI (blue) and quantifications (right) of **a** aYAP1 intensity (red), **b** Yy1 intensity (red) after transduction of 1E+13 vg/kg or 2E+13 vg/kg dose for (**a)** a*YAP1* or (**b)** *Yy1* in control or *Lmna* DCM mice, n = 5, Brown-Forsythe and Welch ANOVA test with Dunnett’s T3 correction, scale bar = 10 µm. Arrows indicate Yap1 (**a)** or Yy1 (**b)** positive CMs. **Figure S11.** Long-term cardiac performance of *Lmna* DCM supplemented with *Lamin C* or *Sun1* shRNA. (**a** and **b)** Evaluation of long-term cardiac performance by echocardiography in *Lmna* DCM mice supplemented with (**a)** Lamin C or (**b)** *Sun1* shRNA, n = 10. **Figure S12.** Sun1 distribution and nuclear protrusion in *Lmna* DCM. (**a)** Western blot of Sun1 protein levels in mouse heart tissues of control or *Lmna* DCM mice. (**b)** Paraffin heart sections of (left) of Sun1 (red), cTnI (green), Lamin A/C (teal) and DAPI (blue) and quantifications (right) of abnormal Sun1 distribution and nuclear protrusion in *Lmna* DCM, n=5, two-tailed, unpaired T-test with Welch correction. Arrows indicate Sun1 positive CMs, white arrows indicate abnormal Sun1 distribution in CMs. (**c)** Paraffin heart sections (left) of PCM1 (red), cTnI (green) and DAPI (blue) and quantifications (right) of nuclear protrusion in control or *Lmna* DCM mice supplemented with control or *Sun1* shRNA, n = 5, Brown-Forsythe and Welch ANOVA test with Dunnett’s T3 correction, scale bar = 10 µm. **Figure S13.** Nesprin1 distribution and nuclear shape in *Lmna* DCM. (**a)** Quantitative real-time PCR analyses of *Syne1* (Nesprin1) in control or *Lmna* DCM mice, n = 5, two-tailed, unpaired T-test with Welch correction. (**b)** Western blot of Nesprin1 protein levels in mouse heart tissues of control or *Lmna* DCM mice. (**c)** Paraffin heart sections of Sun1 (red), Nesprin1 (teal), cTnI (green) and DAPI (blue) positive cells, scale bar = 10 µm. (**d)** Paraffin heart sections of (left) of Nesprin1 (red), cTnI (green) and DAPI (blue) and quantifications (right) of abnormal Nesprin1 distribution and nuclear protrusion in *Lmna* DCM mice, n = 5, two-tailed, unpaired T-test with Welch correction, scale bar = 10 µm. Arrows indicate Nesprin1 positive CMs, white arrows indicate abnormal Nesprin1 distribution in CMs. **Figure S14.** *KASH* domain suppresses *Lmna* DCM and cardiac fibrosis. (**a** and **b)** SR and H&E staining of paraffin heart sections and quantifications of *Lmna* DCM mice supplemented with control or *KASH* domain. Quantification of myocardial fibrosis of SR sections, n = 5, Brown-Forsythe and Welch ANOVA test with Dunnett’s T3 correction. For complete heart images: magnification = 4 ×, scale bar =1000 µm; for enlarged images: magnification = 20 ×, scale bar = 100 µm. (**c)** Quantitative real-time PCR analyses of *Nppa*, *Nppb*, *Col1a1* and *Col1a2, KASH* domain in *Lmna* DCM mice supplemented with control or *KASH* domain, n = 5, Brown-Forsythe and Welch ANOVA test with Dunnett’s T3 correction. **Figure S15.** Effect of DNSUN1 on inducible *Lmn*a DCM mice. (**a)** Survival curve of inducible *Lmna* DCM mice treated with DNSUN1 (Red) at a dose of 1E+14 vg/kg compared to inducible *Lmna* DCM mice (Black) and Ctrl (Blue), n ≥ 5, Log- rank (Mantel- Cox) test. **Figure S16.** *LMNA* Mutations. Schematic diagram of *LMNA* mutations depicted by green lines located along the Prelamin A and Lamin A/C. Diagram is adapted from Broers et al [63].

**Additional file 2: Table S1.** Effect of potential candidates in wildtype mice. Echocardiography of potential candidates Lamin A at a dose of 1E+13 vg/kg assessed at 5.5 weeks. P value represents comparisons to *EGFP* control, two-tailed, unpaired T-test with Welch correction and Mann-Whitney test. **Table S2.** Effect of Lamin A on wildtype mice. Effect of Lamin A at a dose of 2E+13 vg/kg assessed by echocardiography at 5.5 weeks. P value represents comparisons to *EGFP* control, two-tailed, unpaired T-test with Welch correction. **Table S3.** Effect of cardiac specific upregulation of mature Lamin A. Effect of mature Lamin A at a dose of 2E+13 vg/kg assessed by echocardiography at 5.5 weeks. P value represents comparisons to *EGFP* control, two-tailed, unpaired T-test with Welch correction. **Table S4.** Effect of potential candidates on *Lmna* DCM in mice. Echocardiography of *Lmna* DCM mice supplemented with control or potential candidates *Sun1* shRNA, *Bmp7-Ctgf* shRNA, Lamin C, a*YAP1*, Lamin A, *Smad3* shRNA, *Yy1*, *Ctgf *shRNA, *Bmp7*, *Fgf16*, *Smad2* shRNA, *Mapk14* shRNA, *Yap1* shRNA, *Tgfb1* shRNA, *Raptor* shRNA or *Serca2a* at a dose of 1E+13 vg/kg assessed at 5.5 weeks. P value represents comparisons to *Lmna* DCM, Brown-Forsythe and Welch ANOVA test with Dunnett’s T3 correction and Kruskal- Wallis test. LVDD, left ventricular diastolic dimension; LVWT, LV wall thickness; EF, ejection fraction; FS, fractional shortening. *One mouse died before echocardiography. **Table S5.** Effect of potential candidates in wildtype mice. Echocardiography of potential candidates Lamin C, *Sun1*, *Bmp7*-*Ctgf *shRNA, a*YAP1*, *Smad3* shRNA, *Yy1*, *Ctgf* shRNA, *Bmp7*, *Fgf16*, *Smad2* shRNA, *Mapk14* shRNA, *Yap1* shRNA, *Tgfb1* shRNA, *Raptor* shRNA or *Serca2a* or control at a dose of 1E+13 vg/kg assessed at 5.5 weeks. P value represents comparisons to control, Brown-Forsythe and Welch ANOVA test with Dunnett’s T3 correction. **Table S6.** Effect of selected candidates at higher dose on *Lmna* DCM mice. Echocardiography of *Lmna* DCM mice supplemented with control or candidates *Smad2* shRNA, *Yap1* shRNA, *Tgfb1* shRNA, *Mapk14* shRNA, *Smad3* shRNA,* Sun1 *shRNA, *Yy1*, a *YAP1* or control at a dose of 2E+13 vg/kg assessed at 5.5 weeks. P value represents comparisons to control, Brown-Forsythe and Welch ANOVA test with Dunnett’s T3 correction. *One mouse died before echocardiography. **Table S7.** Effect of KASH Domain on *Lmna* DCM Effect of *KASH domain* at a dose of 1.0E+13 vg/kg on *Lmna* DCM mice at 5.5 weeks. P value represented comparisons *Lmna* DCM + Ctrl, Brown-Forsythe and Welch ANOVA test with Dunnett’s T3 correction. LVDD, left ventricular diastolic dimension; LVWT, LV wall thickness; EF, ejection fraction; FS, fraction shortening. **Table S8.** Effect of DNSUN1 on inducible *Lmna* DCM mice Echocardiography of control and inducible *Lmna* DCM mice performed 2.5 weeks after *Lmna* deletion. P value represents comparisons to control, two-tailed, unpaired T-test with Welch correction and Mann-Whitney test. LVDD, left ventricular diastolic dimension; LVWT, LV wall thickness; EF, ejection fraction; FS, fractional shortening. Echocardiography performed on a Prospect T1 ultrasound.

## Data Availability

The datasets during and/or analysed during the current study available from the corresponding author on reasonable request.

## Abbreviations

LMNA: Lamin A/C gene
HF: Heart failure
DCM: Dilated cardiomyopathy
rAAV: Recombinant adeno-associated virus
SMA: Spinal muscular atrophy
IACUC: Institutional animal care and use committee
NACLAR: Guidelines on the care and use of animals for scientific purposes
LVDD: Left ventricular diastolic dimensions
LVWT: Left ventricular wall thickness
EF: Ejection fraction
FS: Fractional shortening
GSEA: Gene set enrichment analysis
qPCR: Quantitative real-time PCR
iPSc: Induced pluripotent stem cells
LINC: Linkers of nucleoskeleton and cytoskeleton
CM: Cardiomyocytes
MI: Myocardial infarction
TAC: Transverse aortic constriction
Tgfβ: Transforming growth factor beta
Fgf: Fibroblast growth factors
Mapk: Mitogen-activated protein kinases
Yy1: Yin Yang 1
Bmp7: Bone morphogenetic protein 7
Ctgf: Connective tissue growth factor
aYAP1: Activated yes-associated protein 1
Sun1: Sad1 and UNC84 domain containing 1
Pdgf: Platelet-derived growth factor
mTor: Mammalian target of rapamycin
BET: Bromodomain and extraterminal
MHC: Major histocompatibility complex
MYBPC3: Myosin binding protein C3
MYL2: Myosin light chain 2
KASH: Klarsicht, ANC-1, Syne homology
Nppa: Natriuretic peptide A
Nppb: Natriuretic peptide B
Myh7: Myosin heavy chain 7
Atp2a2: Sarcoplasmic/endoplasmic reticulum calcium ATPase 2 (Serca2)
Ctcf: CCCTC-binding factor
αSMA: Actin alpha 2, smooth muscle
CD3: Cluster of differentiation 3
Iba1: Allograft inflammatory factor 1 (Aif1)
PCM1: Pericentriolar material 1
γH2AX: Gamma-H2A.X variant histone
cTnI: Cardiac Troponin I
PCSk9: Proprotein convertase subtilisin/kexin type 9
