# Supplementary material for: Systematic in vivo candidate evaluation uncovers therapeutic targets for LMNA dilated cardiomyopathy and risk of Lamin A toxicity
Source: J Transl Med. 2023 Oct 16;21:690. doi: 10.1186/s12967-023-04542-4 (PMC10577912; doi:10.1186/s12967-023-04542-4)
Supplement: Supplementary file 1 — Additional file 1: Figure S1. Upregulation of Lamin A and Lamin C in Lmna DCM mice. Evaluation of the upregulated level of Lamin A and Lamin C in vivo by western blot in mouse whole heart tissue lysates of control or Lmna DCM groups with control, Lamin A or Lamin C upregulation. Figure S2. Upregulation of Lamin A in mice. (a) Experimental timeline showing timepoints of virus injection and echocardiogram. Cardiac performance was assessed by echocardiogram at 5.5 weeks-old. SR and H&E staining of paraffin heart sections 3-4 weeks after transduction of EGFP control or Lamin A. Quantification of myocardial fibrosis of SR sections, virus dose, 1.0E+13 vg/kg, n = 5, Mann-Whitney test. For complete heart images: magnification = 4 ×, scale bar =1000 µm; for enlarged images: magnification = 20 ×, scale bar = 100 µm. (b, c) Quantitative real-time PCR analyses of Nppa, Nppb, Col1a1 and Col1a2 in mice transduced with EGFP control or Lamin A, n = 5, two-tailed, unpaired T-test with Welch correction and Mann-Whitney test. Figure S3. Upregulation of mature Lamin A by AAV leads to DCM and cardiac fibrosis. (a) SR and H&E staining of paraffin heart sections and quantifications of mice transduced with EGFP control or mature Lamin A. Quantification of myocardial fibrosis of SR sections, virus dose, 2.0E+13 vg/kg, n = 5, two-tailed, unpaired T-test with Welch correction. For complete heart images: magnification = 4 ×, scale bar =1000 µm; for enlarged images: magnification = 20 ×, scale bar = 100 µm. (b, c) Quantitative real-time PCR analyses of Nppa, Nppb, Col1a1 and Col1a2 in mice transduced with EGFP control or mature Lamin A, n = 5, two-tailed, unpaired T-test with Welch correction. (d) Western blot of p-Smad2 protein levels in mouse heart tissues of mice transduced with EGFP control or mature Lamin A, n = 5, two-tailed, unpaired T-test with Welch correction. (e–g) Paraffin heart sections (left) and quantifications (right) of (e) αSMA (red), (f) Iba-1 (red), (g) CD3 (red), cTnI (gre [file 12967_2023_4542_MOESM1_ESM.pdf]

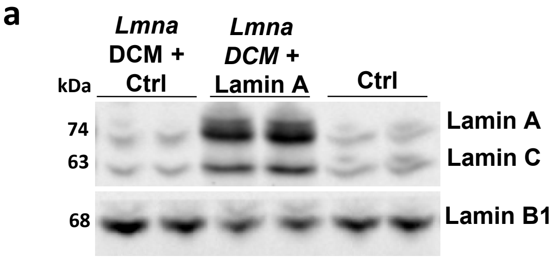

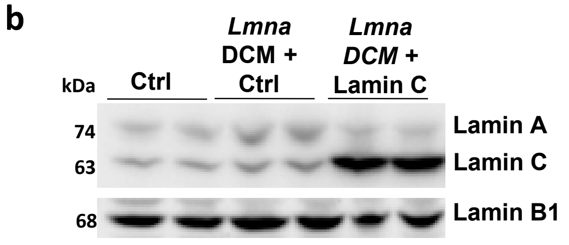

Additional Figure S1

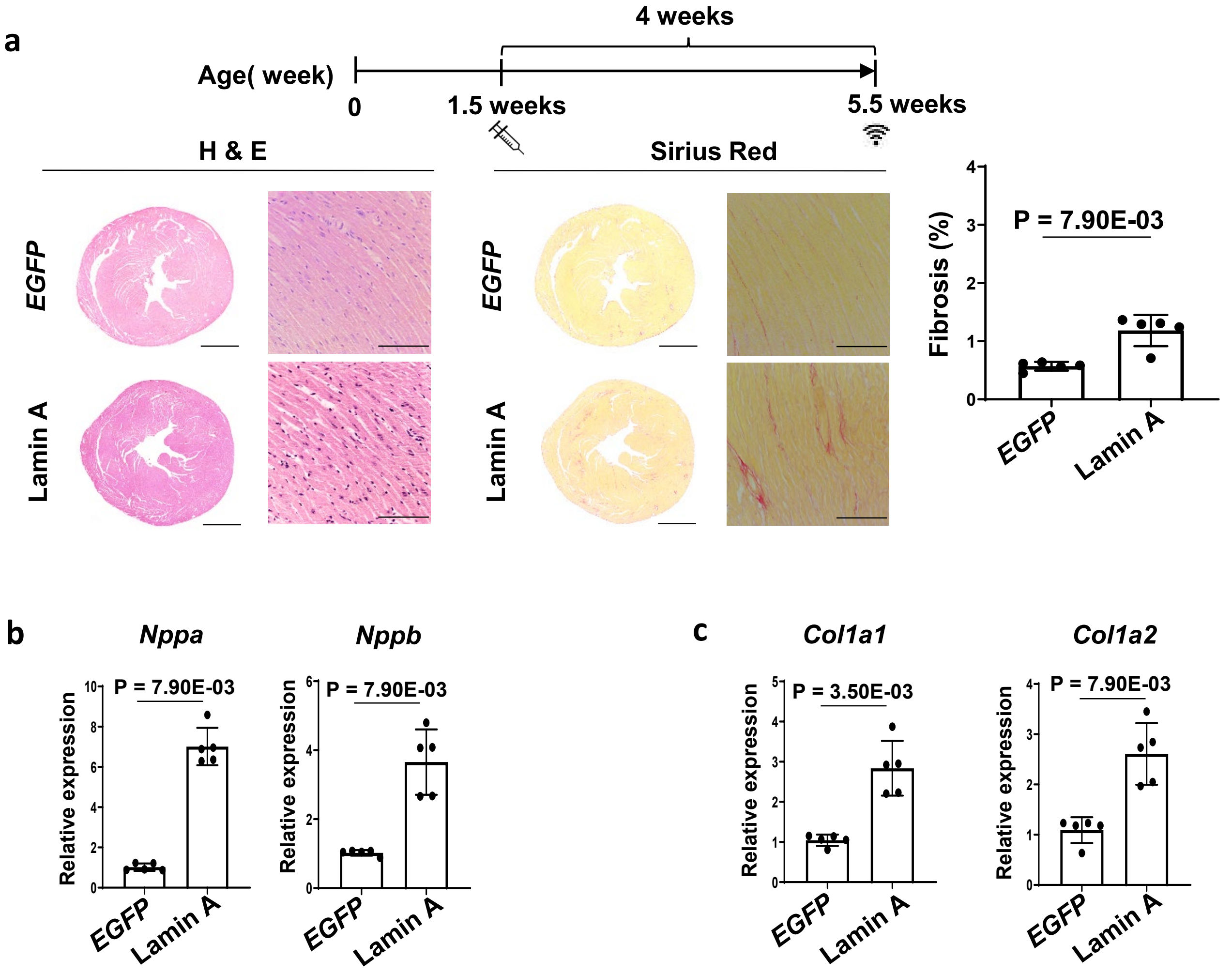

Additional Figure S2

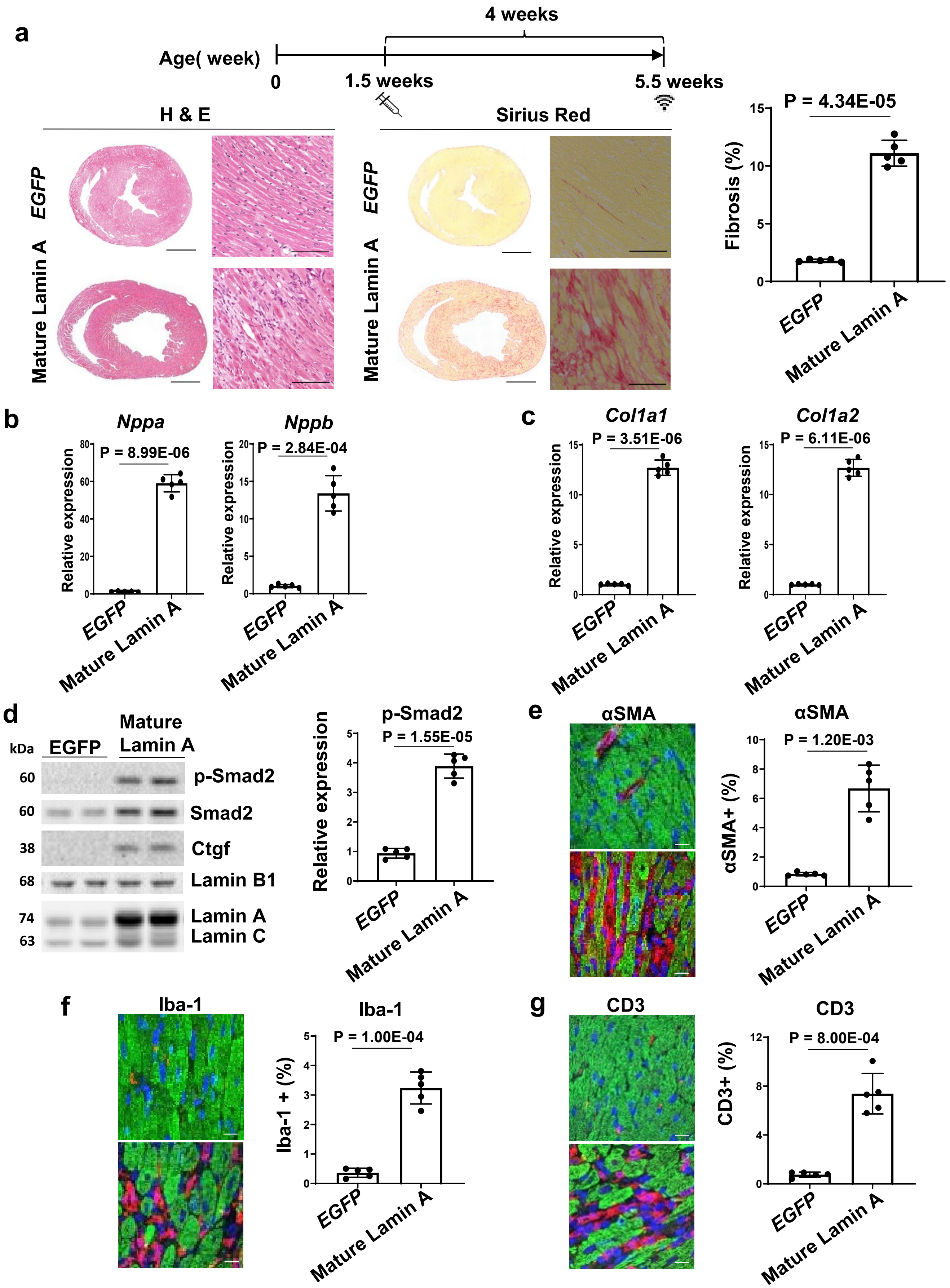

Additional Figure S3

a

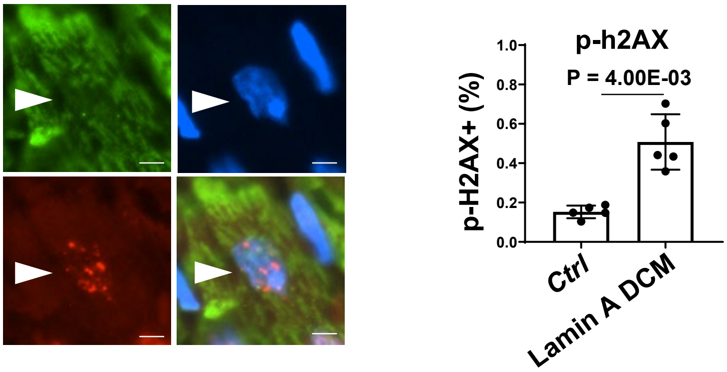

Additional Figure S4

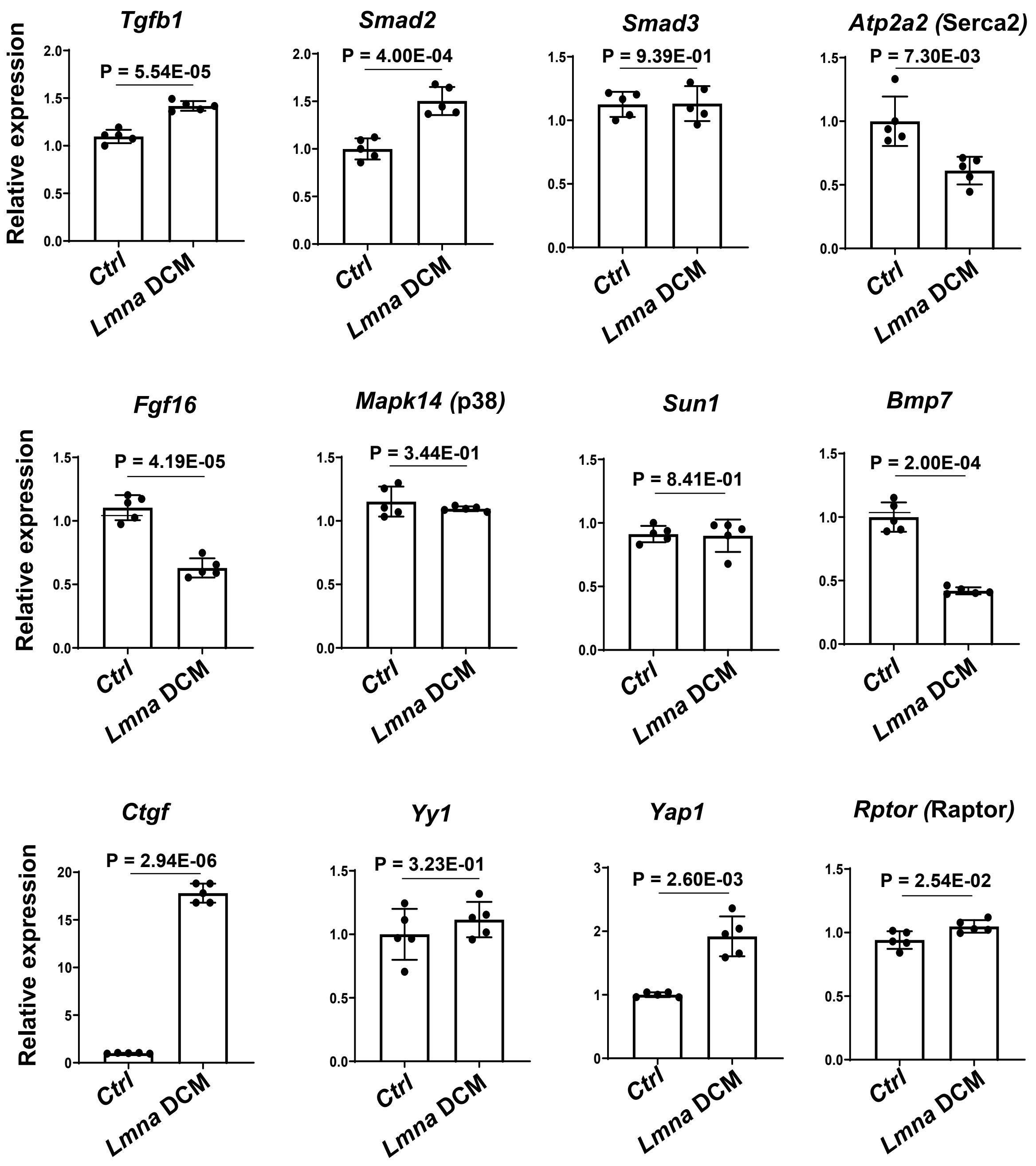

Additional Figure S5

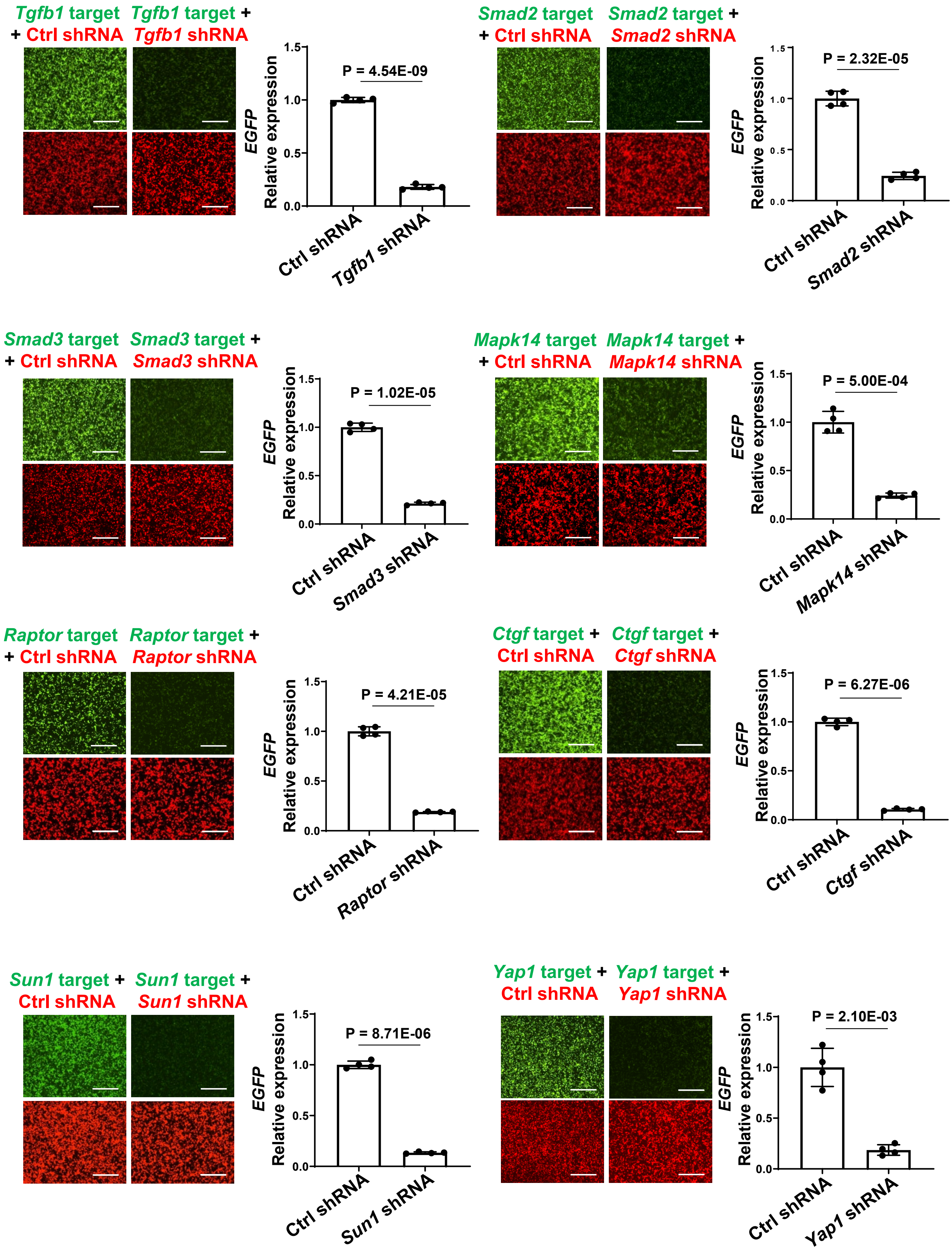

Additional Figure S6

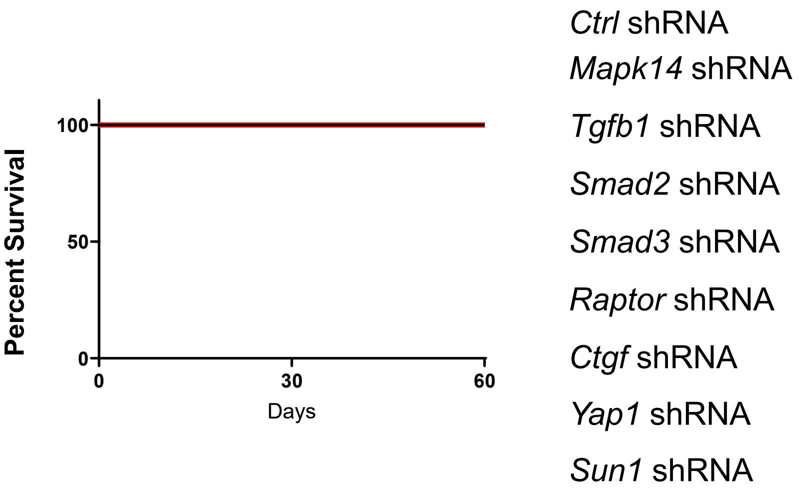

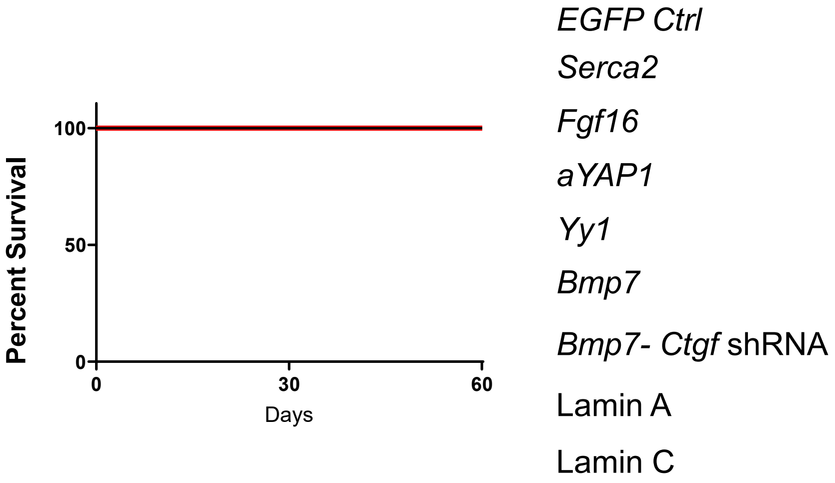

Additional Figure S7

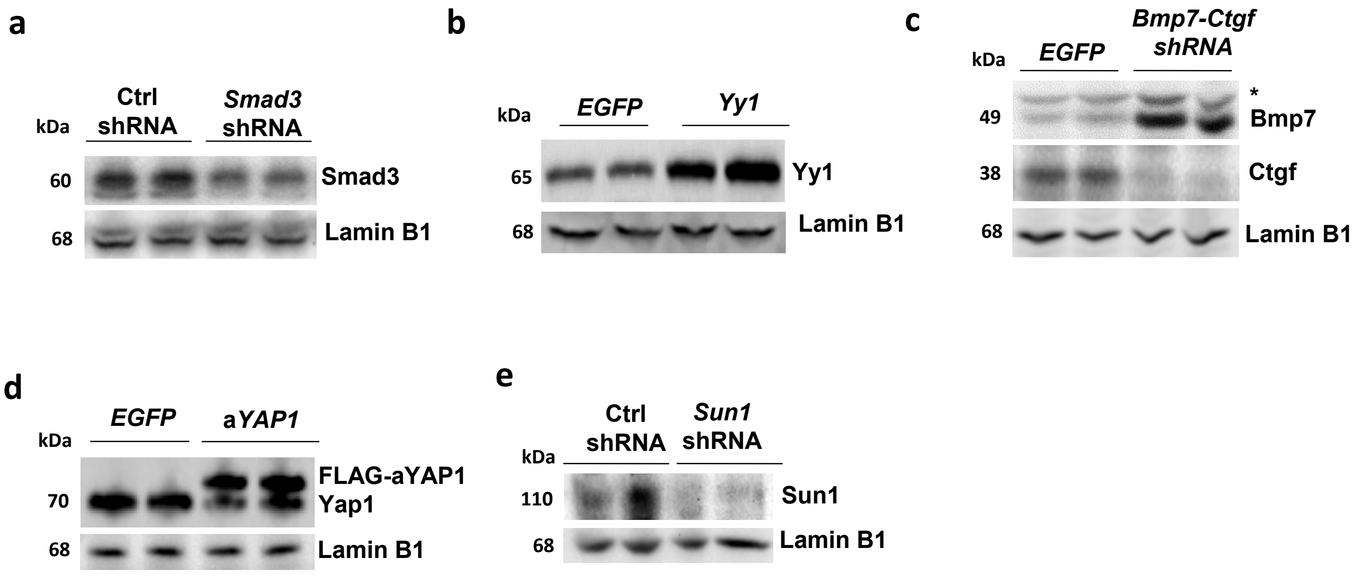

Additional Figure S8

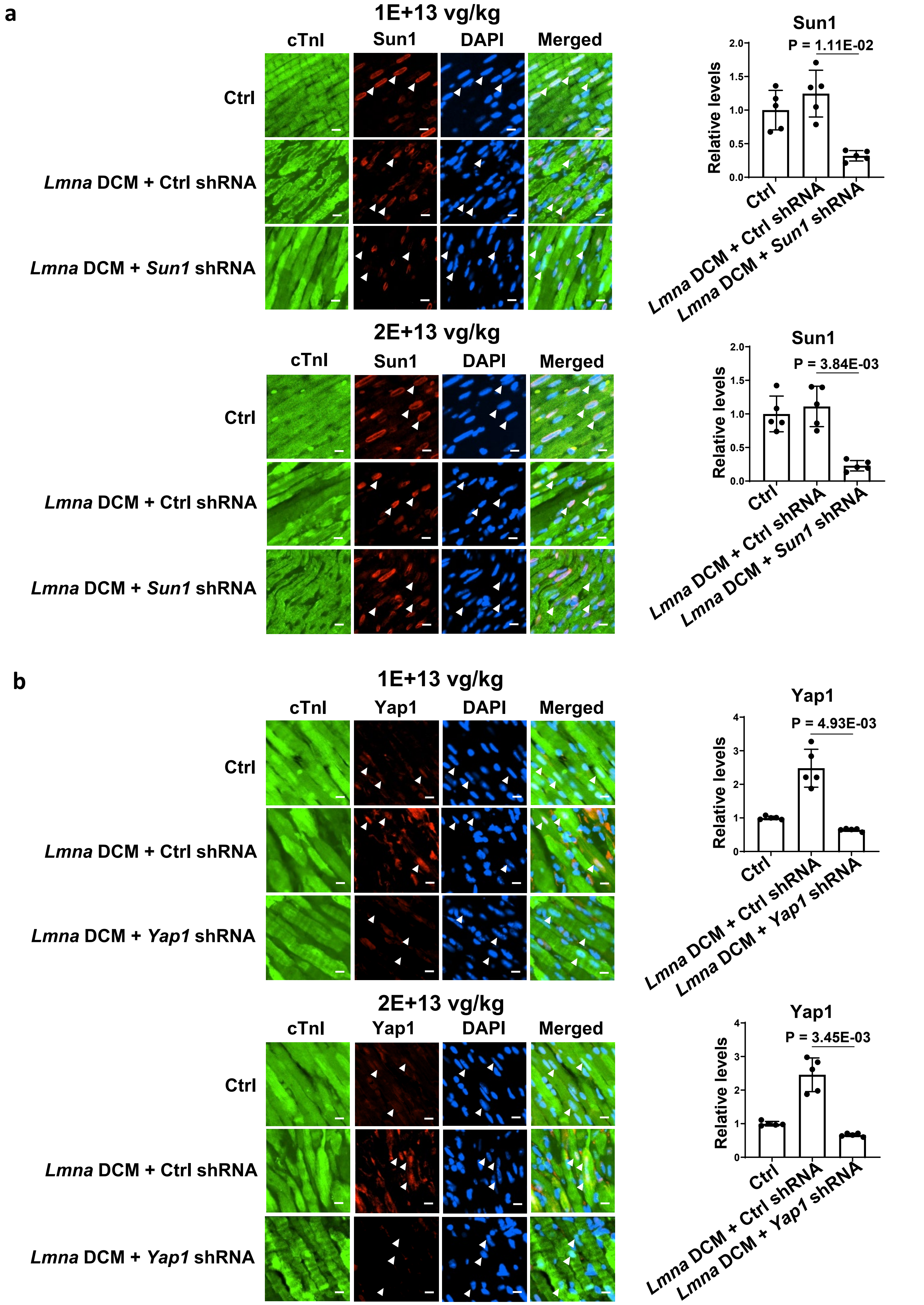

Additional Figure S9

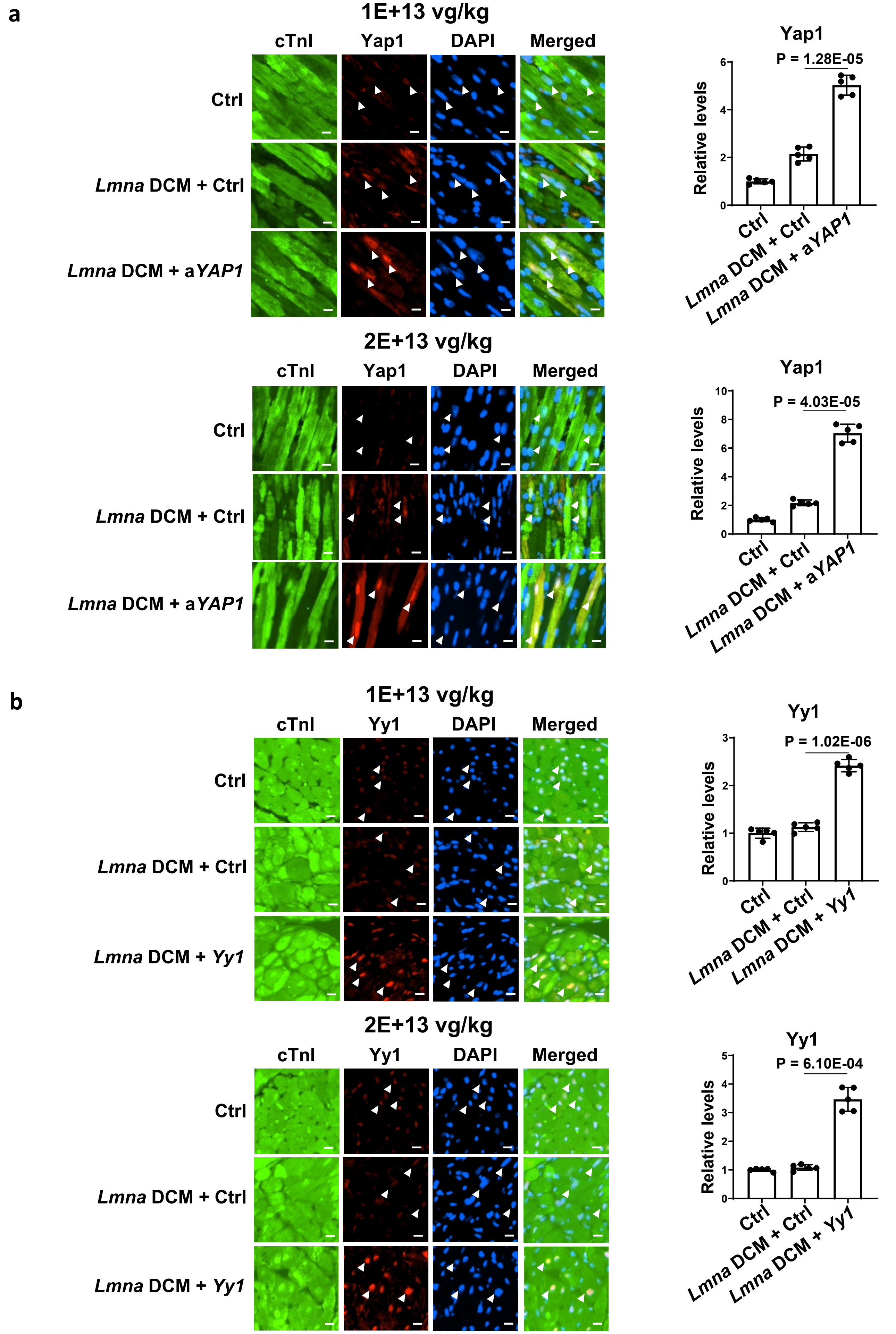

Additional Figure S10

a

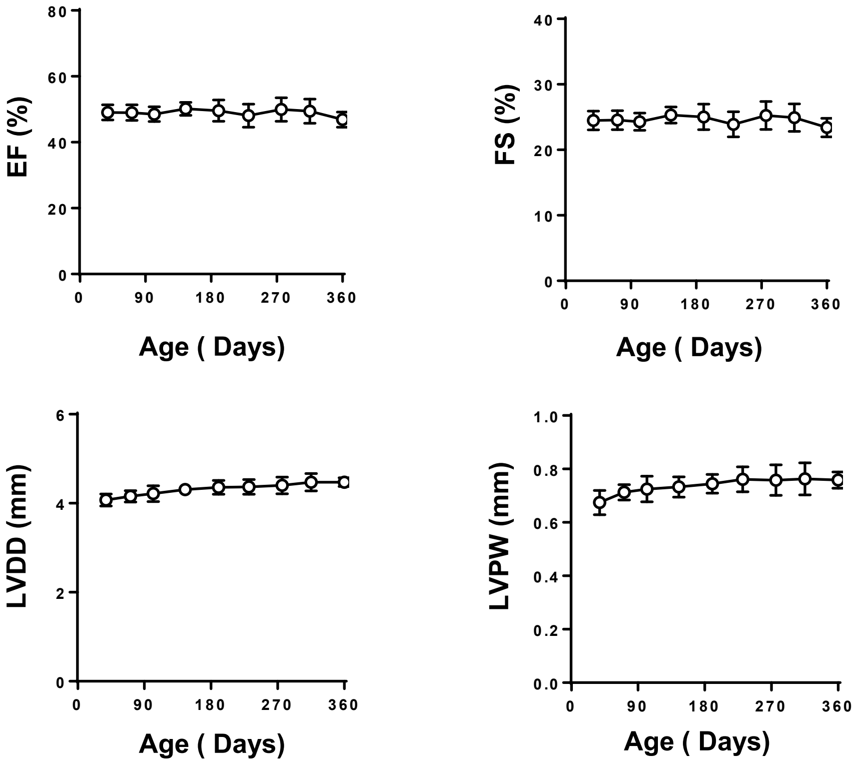

b

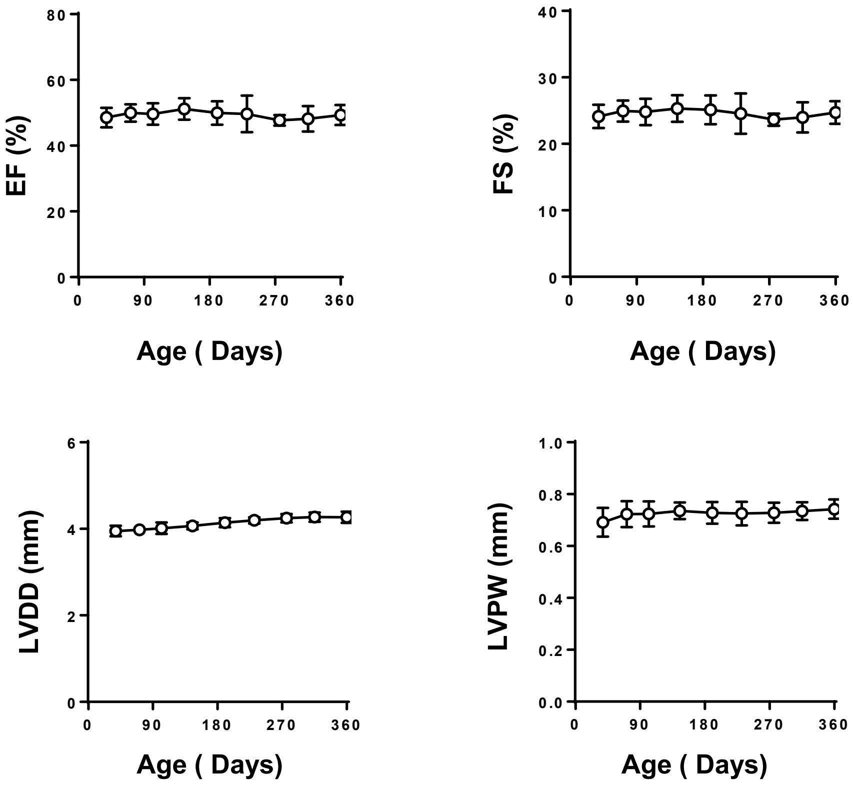

Additional Figure S11

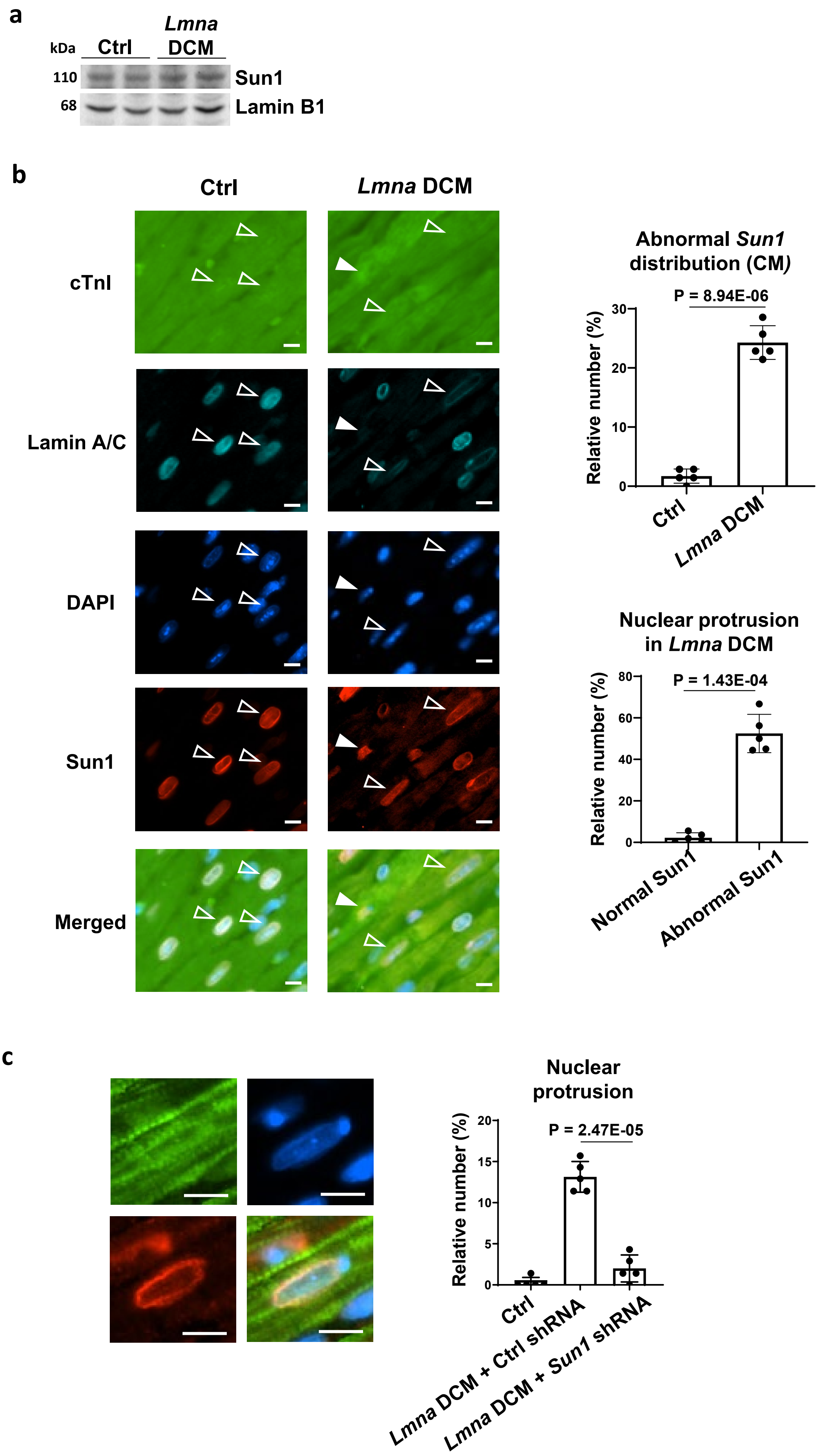

Additional Figure S12

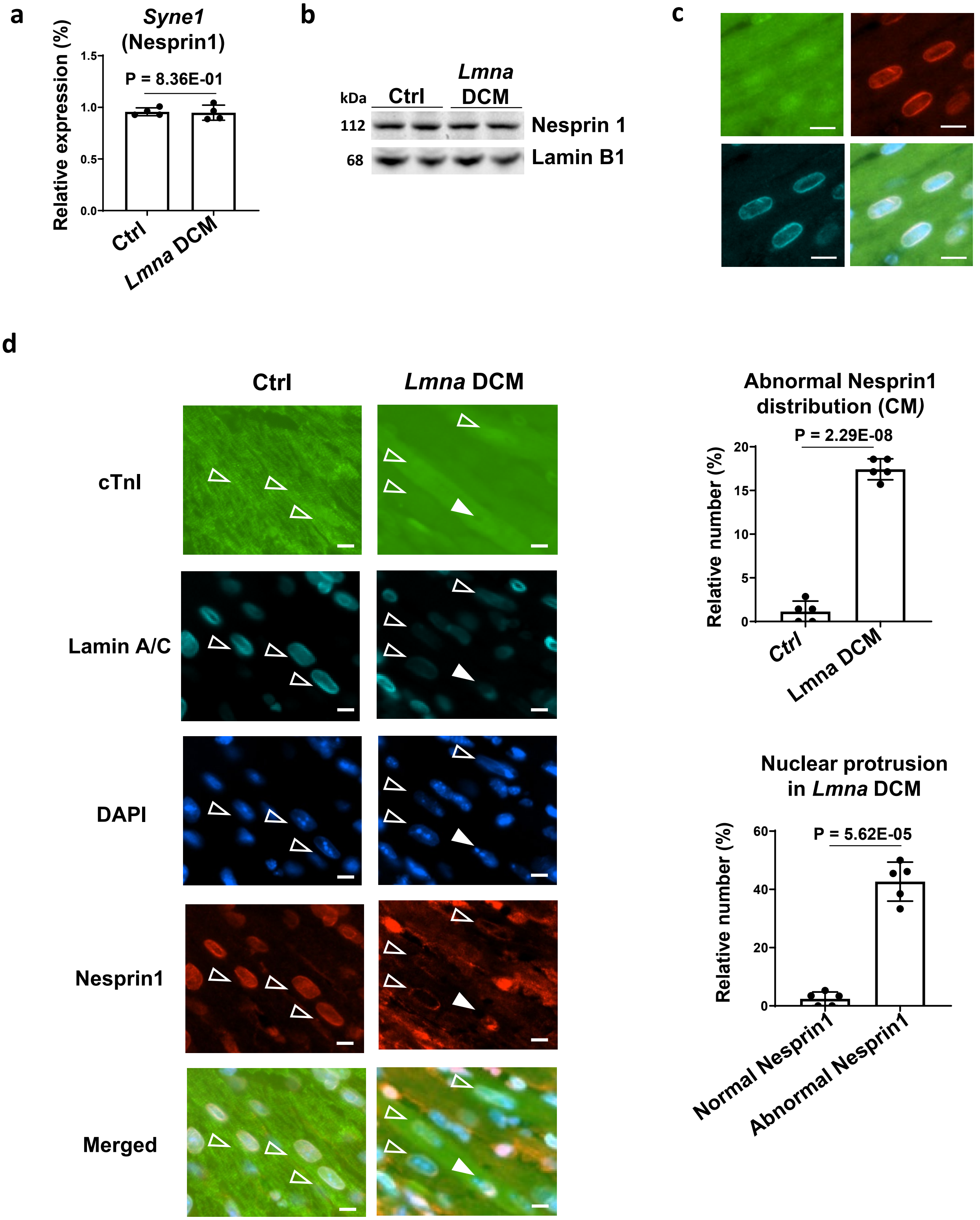

Additional Figure S13

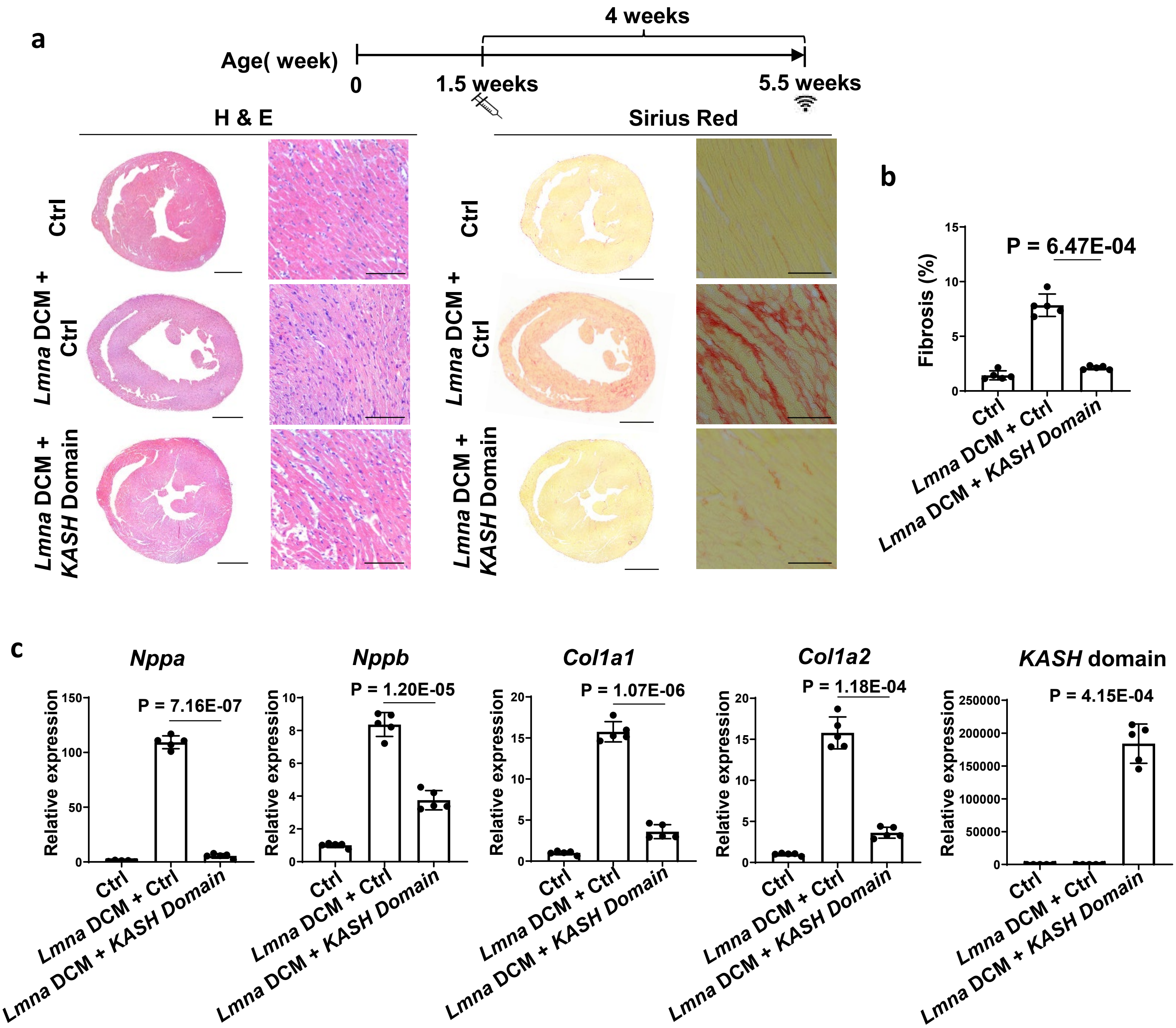

Additional Figure S14

a

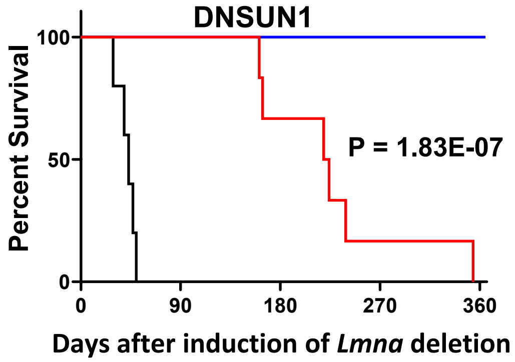

Additional Figure S15

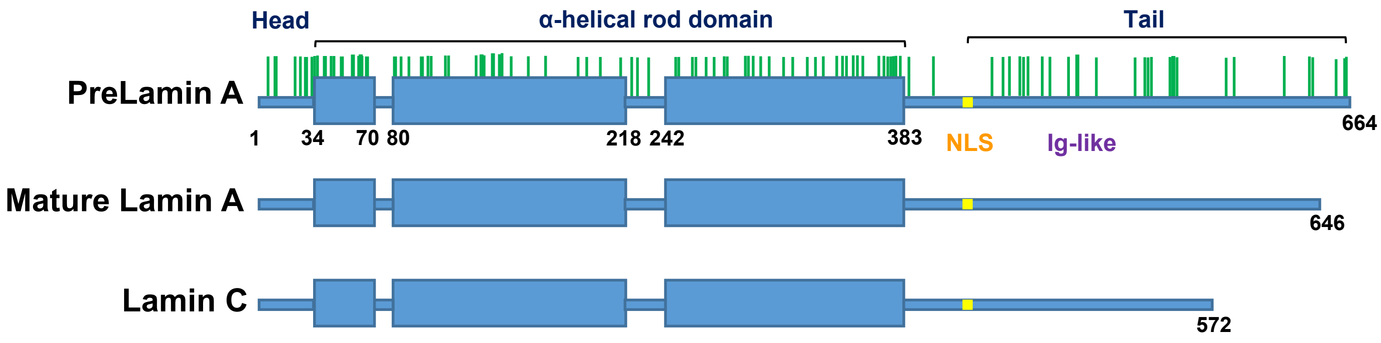

Additional Figure S16
