# Supplementary material for: Systematic in vivo candidate evaluation uncovers therapeutic targets for LMNA dilated cardiomyopathy and risk of Lamin A toxicity
Source: J Transl Med. 2023 Oct 16;21:690. doi: 10.1186/s12967-023-04542-4 (PMC10577912; doi:10.1186/s12967-023-04542-4)
Supplement: Supplementary file 2 — Additional file 2: Table S1. Effect of potential candidates in wildtype mice. Echocardiography of potential candidates Lamin A at a dose of 1E+13 vg/kg assessed at 5.5 weeks. P value represents comparisons to EGFP control, two-tailed, unpaired T-test with Welch correction and Mann-Whitney test. Table S2. Effect of Lamin A on wildtype mice. Effect of Lamin A at a dose of 2E+13 vg/kg assessed by echocardiography at 5.5 weeks. P value represents comparisons to EGFP control, two-tailed, unpaired T-test with Welch correction. Table S3. Effect of cardiac specific upregulation of mature Lamin A. Effect of mature Lamin A at a dose of 2E+13 vg/kg assessed by echocardiography at 5.5 weeks. P value represents comparisons to EGFP control, two-tailed, unpaired T-test with Welch correction. Table S4. Effect of potential candidates on Lmna DCM in mice. Echocardiography of Lmna DCM mice supplemented with control or potential candidates Sun1 shRNA, Bmp7-Ctgf shRNA, Lamin C, aYAP1, Lamin A, Smad3 shRNA, Yy1, Ctgf shRNA, Bmp7, Fgf16, Smad2 shRNA, Mapk14 shRNA, Yap1 shRNA, Tgfb1 shRNA, Raptor shRNA or Serca2a at a dose of 1E+13 vg/kg assessed at 5.5 weeks. P value represents comparisons to Lmna DCM, Brown-Forsythe and Welch ANOVA test with Dunnett’s T3 correction and Kruskal- Wallis test. LVDD, left ventricular diastolic dimension; LVWT, LV wall thickness; EF, ejection fraction; FS, fractional shortening. *One mouse died before echocardiography. Table S5. Effect of potential candidates in wildtype mice. Echocardiography of potential candidates Lamin C, Sun1, Bmp7-Ctgf shRNA, aYAP1, Smad3 shRNA, Yy1, Ctgf shRNA, Bmp7, Fgf16, Smad2 shRNA, Mapk14 shRNA, Yap1 shRNA, Tgfb1 shRNA, Raptor shRNA or Serca2a or control at a dose of 1E+13 vg/kg assessed at 5.5 weeks. P value represents comparisons to control, Brown-Forsythe and Welch ANOVA test with Dunnett’s T3 correction. Table S6. Effect of selected candidates at higher dose on Lmna DCM mice. Echocardiography of Lmna DCM mice supplemented with [file 12967_2023_4542_MOESM2_ESM.docx]

| **Age** | **Virus** | **N** | **LVDD** | **P** | **LVWT** | **P** | **EF%** | **P** | **FS%** | **P** |
| --- | --- | --- | --- | --- | --- | --- | --- | --- | --- | --- |
| 5.5  weeks | *EGFP* | 5 | 3.91±0.09 |  | 0.70±0.06 |  | 55.10±3.54 |  | 28.27±2.26 |  |
|  | Lamin A | 5 | 3.89±0.12 | >9.99E-01 | 0.69±0.02 | 8.16E-01 | 51.59±1.00 | 9.02E-02 | 25.97±0.61 | 8.46E-02 |

**Table S1. Effect of potential candidates in wildtype mice.** Echocardiography of potential candidates Lamin A at a dose of 1E+13 vg/kg assessed at 5.5 weeks. P value represents comparisons to *EGFP* control, two-tailed, unpaired T-test with Welch correction and Mann-Whitney test.

| **Age** | **Virus** | **N** | **LVDD** | **P** | **LVWT** | **P** | **EF%** | **P** | **FS%** | **P** |
| --- | --- | --- | --- | --- | --- | --- | --- | --- | --- | --- |
| 5.5  weeks | *EGFP* | 5 | 3.89±0.06 |  | 0.70±0.05 |  | 55.18±1.59 |  | 28.18±1.06 |  |
|  | Lamin A | 5 | 4.13±0.19 | 4.87E-02 | 0.53±0.04 | 9.00E-04 | 20.53±5.91 | 9.75E-05 | 9.27±2.88 | 3.29E-05 |

**Table S2. Effect of Lamin A on wildtype mice.** Effect of Lamin A at a dose of 2E+13 vg/kg assessed by echocardiography at 5.5 weeks. P value represents comparisons to *EGFP* control, two-tailed, unpaired T-test with Welch correction.

| **Age** | **Virus** | **N** | **LVDD** | **P** | **LVWT** | **P** | **EF%** | **P** | **FS%** | **P** |
| --- | --- | --- | --- | --- | --- | --- | --- | --- | --- | --- |
| 5.5  weeks | *EGFP* | 5 | 3.89±0.15 |  | 0.70±0.06 |  | 53.26±1.22 |  | 27.06±0.86 |  |
|  | Mature  Lamin A | 5 | 4.16±0.19 | 3.63E-02 | 0.54±0.05 | 5.20E-03 | 18.96±6.86 | 2.28E-04 | 8.56±3.35 | 1.12E-04 |

**Table S3. Effect of cardiac specific upregulation of mature Lamin A.** Effect of mature Lamin A at a dose of 2E+13 vg/kg assessed by echocardiography at 5.5 weeks. P value represents comparisons to *EGFP* control, two-tailed, unpaired T-test with Welch correction.

| **Age** | **Virus** | **N** | **LVDD** | **P** | **LVWT** | **P** | **EF%** | **P** | **FS%** | **P** |
| --- | --- | --- | --- | --- | --- | --- | --- | --- | --- | --- |
| 5.5 weeks | *Ctrl* | 20 | 3.91±0.13 |  | 0.67±0.05 |  | 53.66±4.23 |  | 27.34±2.70 |  |
|  | *Lmna* DCM  + *Ctrl* | 20 | 4.30±0.16 |  | 0.53±0.04 |  | 17.63±2.19 |  | 7.87±1.03 |  |
|  | *Lmna* DCM  + *Sun1* shRNA | 5 | 4.03±0.17 | 4.33E-01 | 0.67±0.09 | 3.66E-01 | 47.46±2.00 | 1.03E-05 | 23.50±1.22 | 9.67E-05 |
|  | *Lmna* DCM  +*Bmp7- Ctgf shRNA* | 5 | 3.85±0.10 | 1.67E-03 | 0.67±0.04 | 1.31E-02 | 46.61±4.24 | 9.22E-04 | 22.94±2.49 | 4.65E-03 |
|  | *Lmna* DCM  + Lamin C | 5 | 3.97±0.15 | 1.38E-01 | 0.67±0.03 | 6.34E-03 | 46.68±3.20 | 2.61E-04 | 23.03±2.00 | 5.61E-04 |
|  | *Lmna* DCM  + *aYAP1* | 5 | 3.94±0.08 | 7.18E-04 | 0.66±0.04 | 2.92E-02 | 45.93±3.31 | 2.69E-04 | 22.56±1.85 | 4.63E-04 |
|  | *Lmna* DCM  + Lamin A | 5 | 3.98±0.24 | 6.23E-01 | 0.62±0.02 | 6.96E-03 | 39.41±4.38 | 4.17E-03 | 18.88±2.46 | 1.46E-02 |
|  | *Lmna* DCM  + *Smad3* shRNA | 5 | 4.03±0.05 | 3.13E-03 | 0.62±0.03 | 2.81E-01 | 37.26±6.71 | 1.90E-02 | 17.82±3.66 | 2.40E-02 |
|  | *Lmna* DCM  + *Yy1* | 5 | 3.99±0.03 | 6.20E-03 | 0.61±0.04 | 2.51E-01 | 28.36±5.20 | 4.48E-02 | 13.05±2.64 | 8.20E-02 |
|  | *Lmna* DCM  + *Ctgf* shRNA | 5 | 4.10±0.08 | 1.21E-01 | 0.49±0.06 | 8.16E-01 | 21.82±4.37 | 8.86E-01 | 9.84±2.10 | 8.89E-01 |
|  | *Lmna* DCM  + *Bmp7* | 5 | 4.08±0.35 | 9.96E-01 | 0.52±0.09 | >9.99E-01 | 21.57±4.20 | 9.01E-01 | 9.71±1.98 | 9.07E-01 |
|  | *Lmna* DCM  + *Fgf16* | 5 | 4.05±0.18 | 5.77E-01 | 0.53±0.05 | >9.99E-01 | 21.21±3.26 | 9.96E-01 | 9.55±1.60 | 9.98E-01 |
|  | *Lmna* DCM  + *Smad2* shRNA | 5 | 4.35±0.26 | >9.99E-01 | 0.61±0.02 | 3.35E-04 | 20.86±6.48 | 9.99E-01 | 9.48±3.13 | 9.99E-01 |
|  | *Lmna* DCM  + *Mapk14* shRNA | 5 | 4.26±0.19 | >9.99E-01 | 0.59±0.02 | 6.68E-02 | 20.35±3.67 | 9.85E-01 | 8.97±1.35 | 9.73E-01 |
|  | *Lmna* DCM  + *Yap1* shRNA | 4* | 4.07±0.10 | 2.81E-01 | 0.58±0.04 | 8.47E-02 | 19.61±3.61 | 9.99E-01 | 8.77±1.67 | 9.99E-01 |
|  | *Lmna* DCM  + *TgfB1* shRNA | 5 | 4.30±0.12 | >9.99E-01 | 0.58±0.05 | 5.86E-01 | 19.22±1.93 | 9.91E-01 | 8.69±0.99 | 9.86E-01 |
|  | *Lmna* DCM  + *Raptor* shRNA | 5 | 4.54±0.13 | 3.42E-01 | 0.53±0.09 | >9.99E-01 | 15.57±4.96 | 9.99E-01 | 6.99±2.31 | 9.99E-01 |
|  | *Lmna* DCM  + *Serca2a* | 5 | 4.22±0.11 | 9.99E-01 | 0.54±0.10 | >9.99E-01 | 14.76±5.58 | 9.99E-01 | 6.55±2.55 | 9.99E-01 |

**Table S4. Effect of potential candidates on *Lmna* DCM in mice**. Echocardiography of *Lmna* DCM mice supplemented with control or potential candidates *Sun1* shRNA, *Bmp7-Ctgf* shRNA, Lamin C, a*YAP1*, Lamin A, *Smad3* shRNA, *Yy1*, *Ctgf* shRNA, *Bmp7*, *Fgf16*, *Smad2* shRNA, *Mapk14* shRNA, *Yap1* shRNA, *Tgfb1* shRNA, *Raptor* shRNA or *Serca2a* at a dose of 1E+13 vg/kg assessed at 5.5 weeks. P value represents comparisons to *Lmna* DCM, Brown-Forsythe and Welch ANOVA test with Dunnett’s T3 correction and Kruskal- Wallis test. LVDD, left ventricular diastolic dimension; LVWT, LV wall thickness; EF, ejection fraction; FS, fractional shortening. *One mouse died before echocardiography.

| **Age** | **Virus** | **N** | **LVDD** | **P** | **LVWT** | **P** | **EF%** | **P** | **FS%** | **P** |
| --- | --- | --- | --- | --- | --- | --- | --- | --- | --- | --- |
| 5.5 weeks | *Ctrl* | 15 | 3.88±0.09 |  | 0.71±0.05 |  | 55.03±1.91 |  | 28.13±1.24 |  |
|  | Lamin C | 5 | 3.88±0.09 | >9.99E-01 | 0.68±0.05 | >9.99E-01 | 57.14±5.13 | 9.99E-01 | 29.64±3.45 | 9.99E-01 |
|  | *Sun1* shRNA | 5 | 3.89±0.16 | >9.99E-01 | 0.69±0.05 | >9.99E-01 | 55.28±2.33 | >9.99E-01 | 28.36±1.51 | >9.99E-01 |
|  | *Bmp7- Ctgf shRNA* | 5 | 3.88±0.03 | >9.99E-01 | 0.68±0.03 | >9.99E-01 | 56.99±0.92 | 3.83E-01 | 29.50±0.60 | 2.82E-01 |
|  | *aYAP1* | 5 | 3.87±0.09 | >9.99E-01 | 0.70±0.07 | >9.99E-01 | 57.24±6.33 | 9.99E-01 | 29.65±4.20 | 9.99E-01 |
|  | *Smad3* shRNA | 5 | 3.87±0.05 | >9.99E-01 | 0.70±0.03 | >9.99E-01 | 54.06±1.55 | 9.99E-01 | 27.43±0.91 | 9.99E-01 |
|  | *Yy1* | 5 | 3.85±0.06 | >9.99E-01 | 0.69±0.01 | >9.99E-01 | 53.12±2.38 | 9.81E-01 | 26.98±1.48 | 9.86E-01 |
|  | *Ctgf* shRNA | 5 | 3.89±0.15 | >9.99E-01 | 0.69±0.02 | >9.99E-01 | 54.13±3.60 | >9.99E-01 | 27.56±2.40 | >9.99E-01 |
|  | *Bmp7* | 5 | 3.87±0.09 | >9.99E-01 | 0.68±0.07 | >9.99E-01 | 57.72±3.36 | 9.64E-01 | 29.89±2.31 | 9.75E-01 |
|  | *Fgf16* | 5 | 3.88±0.04 | >9.99E-01 | 0.70±0.04 | >9.99E-01 | 54.59±3.18 | >9.99E-01 | 27.88±2.09 | >9.99E-01 |
|  | *Smad2* shRNA | 5 | 3.86±0.02 | >9.99E-01 | 0.70±0.03 | >9.99E-01 | 54.80±2.22 | >9.99E-01 | 27.93±1.47 | >9.99E-01 |
|  | *Mapk14* shRNA | 5 | 3.86±0.09 | >9.99E-01 | 0.69±0.11 | >9.99E-01 | 56.50±0.86 | 8.10E-01 | 29.03±0.56 | 8.86E-01 |
|  | *Yap1* shRNA | 5 | 3.87±0.04 | >9.99E-01 | 0.70±0.06 | >9.99E-01 | 53.75±2.75 | 9.99E-01 | 27.33±1.78 | 9.99E-01 |
|  | *TgfB1* shRNA | 5 | 3.88±0.02 | >9.99E-01 | 0.70±0.02 | >9.99E-01 | 53.53±1.58 | 9.75E-01 | 27.15±0.99 | 9.69E-01 |
|  | *Raptor* shRNA | 5 | 3.87±0.04 | >9.99E-01 | 0.69±0.02 | >9.99E-01 | 54.61±1.83 | >9.99E-01 | 27.84±1.29 | >9.99E-01 |
|  | *Serca2a* | 5 | 3.85±0.05 | 9.99E-01 | 0.71±0.06 | >9.99E-01 | 53.16±1.49 | 8.26E-01 | 26.79±0.94 | 6.88E-01 |

**Table S5. Effect of potential candidates in wildtype mice.** Echocardiography of potential candidates Lamin C, *Sun1*, *Bmp7*-*Ctgf* shRNA, a*YAP1*, *Smad3* shRNA, *Yy1*, *Ctgf* shRNA, *Bmp7*, *Fgf16*, *Smad2* shRNA, *Mapk14* shRNA, *Yap1* shRNA, *Tgfb1* shRNA, *Raptor* shRNA or *Serca2a* or control at a dose of 1E+13 vg/kg assessed at 5.5 weeks. P value represents comparisons to control, Brown-Forsythe and Welch ANOVA test with Dunnett’s T3 correction.

| **Age** | **Virus** | **N** | **LVDD** | **P** | **LVWT** | **P** | **EF%** | **P** | **FS%** | **P** |
| --- | --- | --- | --- | --- | --- | --- | --- | --- | --- | --- |
| 5.5 weeks | *Ctrl* | 15 | 3.95±0.10 |  | 0.71±0.06 |  | 55.13±2.81 |  | 28.29±1.78 |  |
|  | *Lmna* DCM  + *Ctrl* | 15 | 4.31±0.12 |  | 0.52±0.04 |  | 17.18±1.77 |  | 7.66±0.83 |  |
|  | *Lmna* DCM  *+ Smad2* shRNA | 5 | 4.48±0.16 | 7.01E-01 | 0.60±0.00 | 4.73E-05 | 16.45±4.22 | >9.99E-01 | 7.38±1.95 | >9.99E-01 |
|  | *Lmna* DCM  *+ Yap1* shRNA | 4* | 4.27±0.10 | 9.99E-01 | 0.53±0.02 | 9.99E-01 | 17.56±1.92 | >9.99E-01 | 7.84±0.89 | >9.99E-01 |
|  | *Lmna* DCM  *+ TgfB1* shRNA | 5 | 4.37±0.06 | 9.72E-01 | 0.60±0.03 | 2.45E-02 | 17.09±0.23 | >9.99E-01 | 7.63±0.12 | >9.99E-01 |
|  | *Lmna* DCM  *+ Mapk14* shRNA | 5 | 4.31±0.05 | >9.99E-01 | 0.58±0.10 | 9.84E-01 | 18.28±1.01 | 9.38E-01 | 8.23±0.55 | >9.99E-01 |
|  | *Lmna* DCM  *+ Smad3* shRNA | 5 | 4.10±0.07 | 3.10E-02 | 0.61±0.04 | 4.61E-02 | 32.41±2.80 | 1.72E-03 | 15.17±1.50 | 9.89E-01 |
|  | *Lmna* DCM  *+ Sun1* shRNA | 5 | 3.98±0.05 | 1.13E-05 | 0.68±0.02 | 6.89E-07 | 48.88±3.60 | 1.47E-04 | 24.28±2.22 | 3.60E-02 |
|  | *Lmna* DCM  *+ Yy1* | 5 | 3.99±0.03 | 1.15E-06 | 0.61±0.04 | 5.01E-02 | 28.84±4.89 | 8.30E-02 | 13.29±2.48 | >9.99E-01 |
|  | *Lmna* DCM  *+* a*YAP1* | 5 | 3.96±0.07 | 3.43E-04 | 0.66±0.03 | 3.73E-03 | 45.92±3.14 | 1.28E-04 | 22.56±1.86 | 1.05E-01 |

**Table S6. Effect of selected candidates at higher dose on *Lmna* DCM mice.** Echocardiography of *Lmna* DCM mice supplemented with control or candidates *Smad2* shRNA, *Yap1* shRNA, *Tgfb1* shRNA, *Mapk14* shRNA, *Smad3* shRNA, *Sun1* shRNA, *Yy1*, a*YAP1* or control at a dose of 2E+13 vg/kg assessed at 5.5 weeks. P value represents comparisons to control, Brown-Forsythe and Welch ANOVA test with Dunnett’s T3 correction. *One mouse died before echocardiography.

| **Age** | **Virus** | **N** | **LVDD** | **P** | **LVWT** | **P** | **EF%** | **P** | **FS%** | **P** |
| --- | --- | --- | --- | --- | --- | --- | --- | --- | --- | --- |
| 5.5  weeks | *Ctrl* | 5 | 3.99±0.02 |  | 0.74±0.03 |  | 53.55±1.98 |  | 27.35±1.22 |  |
|  | *Lmna* DCM  + *Ctrl* | 5 | 4.43±0.14 |  | 0.59±0.06 |  | 19.01±4.76 |  | 8.61±2.27 |  |
|  | *Lmna* DCM  + *KASH* Domain | 5 | 4.07±0.06 | 4.86E-03 | 0.69±0.01 | 4.44E-02 | 46.14±3.13 | 4.03E-05 | 22.83±1.84 | 1.30E-05 |

**Table S7. Effect of KASH Domain on *Lmna* DCM** Effect of *KASH domain* at a dose of 1.0E+13 vg/kg on *Lmna* DCM mice at 5.5 weeks. P value represented comparisons *Lmna* DCM + Ctrl, Brown-Forsythe and Welch ANOVA test with Dunnett’s T3 correction. LVDD, left ventricular diastolic dimension; LVWT, LV wall thickness; EF, ejection fraction; FS, fraction shortening.

| **Age** | **Virus** | **N** | **LVDD** | **P** | **LVWT** | **P** | **EF%** | **P** | **FS%** | **P** |
| --- | --- | --- | --- | --- | --- | --- | --- | --- | --- | --- |
| 2.5  weeks post induction | *Ctrl* | 7 | 3.44±0.33 |  | 0.83±0.15 |  | 61.92±4.50 |  | 32.67±3.24 |  |
|  | Inducible *Lmna* DCM | 11 | 3.55±0.30 | 4.88E-01 | 0.72±0.05 | 1.21E-01 | 50.10±9.22 | 2.41E-03 | 25.14±5.58 | 2.31E-03 |

**Table S8. Effect of DNSUN1 on inducible *Lmna* DCM mice** Echocardiography of control and inducible *Lmna* DCM mice performed 2.5 weeks after *Lmna* deletion. P value represents comparisons to control, two-tailed, unpaired T-test with Welch correction and Mann-Whitney test. LVDD, left ventricular diastolic dimension; LVWT, LV wall thickness; EF, ejection fraction; FS, fractional shortening. Echocardiography performed on a Prospect T1 ultrasound.
